# Visualizing water-filled versus embolized status of xylem conduits by desktop x-ray microtomography

**DOI:** 10.1186/1746-4811-9-11

**Published:** 2013-04-08

**Authors:** Jussi-Petteri Suuronen, Marko Peura, Kurt Fagerstedt, Ritva Serimaa

**Affiliations:** 1 Department of Physics, University of Helsinki, P.O.B. 64, Helsinki, FI-00014, Finland; 2 Present address: Central Administration, University of Helsinki, P.O.B. 33, Helsinki, FI-00014, Finland; 3 Department of Biosciences, University of Helsinki, P.O.B. 65, Helsinki, FI-00014, Finland

**Keywords:** Xylem, Cavitation, Birch, Tomography

## Abstract

**Background:**

The hydraulic conductivity of the stem is a major factor limiting the capability of trees to transport water from the soil to transpiring leaves. During drought conditions, the conducting capacity of xylem can be reduced by some conduits being filled with gas, i.e. embolized. In order to understand the dynamics of embolism formation and repair, considerable attention has been given to developing reliable and accurate methods for quantifying the phenomenon. In the past decade, non-destructive imaging of embolism formation in living plants has become possible. Magnetic resonance imaging has been used to visualize the distribution of water within the stem, but in most cases it is not possible to resolve individual cells. Recently, high-resolution synchrotron x-ray microtomography has been introduced as a tool to visualize the water contents of individual cells in vivo, providing unprecedented insight into the dynamics of embolism repair. We have investigated the potential of an x-ray tube -based microtomography setup to visualize and quantify xylem embolism and embolism repair in water-stressed young saplings and shoot tips of Silver and Curly birch (*Betula pendula* and *B. pendula* var. *carelica*).

**Results:**

From the microtomography images, the water-filled versus gas-filled status of individual xylem conduits can be seen, and the proportion of stem cross-section that consists of embolized tissue can be calculated. Measuring the number of embolized vessels in the imaged area is a simple counting experiment. In the samples investigated, wood fibers were cavitated in a large proportion of the xylem cross-section shortly after watering of the plant was stopped, but the number of embolized vessels remained low several days into a drought period. Under conditions of low evaporative demand, also refilling of previously embolized conduits was observed.

**Conclusions:**

Desktop x-ray microtomography is shown to be an effective method for evaluating the water-filled versus embolized status of the stem xylem in a small living sapling. Due to its non-destructive nature, the risk of inducing embolisms during sampling is greatly reduced. Compared with synchrotron imaging beamlines, desktop microtomography offers easier accessibility, while maintaining sufficient resolution to visualize the water contents of individual cells.

## Background

The presently accepted theory explaining the ascent of sap inside a tree stem is the cohesion-tension theory, which is mainly based on four points: the transpirational pull, the xylem forming an interconnected network of conduits, the attractive interactions (hydrogen bonds) between water molecules, and the fact that the water is confined to relatively narrow conduits, remaining in a liquid state even under considerable tensions up to -10 MPa [1].

An important factor limiting the hydraulic conductivity is that water under such high tensions is in a metastable state and vulnerable to cavitation, i.e. formation of gas bubbles within the water column, which will rapidly expand and fill the whole conduit, forming an embolism that prevents the transpirational pull from being transmitted into the tissue below. Drought conditions as well as freeze-thaw cycles experienced by temperate and boreal species pose an increased threat of cavitation for trees [2].

Trees also have means of restoring the hydraulic conductivity lost by cavitation, either by producing new xylem to replace embolized conduits, or by refilling gas-filled cells with water. Under low evaporative demand, many species are able to generate root or stem pressures well above the atmospheric pressure throughout their vascular system [3]. These pressures are thought to be responsible for embolism repair occurring in the spring before leaf flush, and during night time in the growing season [3,4]. Refilling during transpiration is a more complex phenomenon, since it implies the existence of a significant pressure difference between the refilling conduit and the surrounding xylem. This local refilling is not very well understood, but most hypotheses attribute it to hydraulic isolation of the refilling conduit due to the geometry of the pits connecting the xylem elements, and to active secretion of solutes into the embolized conduit by adjacent still-living cells [5,6]. In a recent review, the source of the refilling water was proposed to be the phloem, rather than the water-conducting xylem around the embolized conduits [7].

Until recently, a key limitation in the study of embolism development and repair has been the lack of methods to directly observe cavitation and refilling events in living plants. Traditional methods for quantifying xylem embolism include studying the cut surface of an excised stem or branch segment or leaf petiole under a microscope [8,9], or measuring its percentage loss of hydraulic conductivity (PLC, [6,10]). Also acoustic emissions have been used to detect cavitation events in vivo [11,12]. The former two are destructive methods, and therefore only give the water status of the sample at one specific point in time. With these methods, embolism development and repair can only be observed indirectly, by sampling a group of similar plants undergoing the same environmental changes. Ultrasound observations are a direct method for detecting cavitation as it occurs, but we are unaware of any studies using acoustic emissions to detect refilling, which is presumably a much slower process. Moreover, ultrasound and PLC measurements, respectively, only give information on the number of embolized conduits and amount of conductivity lost due to embolism; the spatial distribution of embolized conduits with in the stem is not accessible with these methods.

The analysis of the effects of water stress on trees and verification of refilling theories would benefit from information on what are the water contents of individual cells. Optical or scanning electron microscopy (SEM) of vitrified tissues (e.g. [13]) could provide the needed spatial information, but both methods are relatively laborious. These methods also involve either freezing the sample rapidly to vitrify the cells’ water contents, or perfusing the excised segment with a staining agent to see which cells are conducting. Combined with cutting the samples, this poses a risk of artificially inducing embolisms during sample preparation (see e.g. [14-16]).

Magnetic resonance imaging (MRI) is one tool capable of non-destructively visualizing the stem water contents, and has already been successfully used to observe embolization in woody species such as grapevines and lianas [17-19]. The disadvantage of MRI is the relatively poor spatial resolution (in-slice resolution of 20-150 μm / pixel), which is adequate to resolve large embolized vessels, but not the surrounding tissue.

Recently, synchrotron-based x-ray imaging has been applied to observing xylem embolism and refilling. Lee and Kim [20] used phase-contrast micro-imaging to observe the refilling of vessels in bamboo, and Brodersen et al. [21] demonstrated the use of synchrotron-based high resolution x-ray microtomography (XMT, also known as μCT) in visualizing embolism refilling in grapevine stems. With a resolution of 4.4 μm per voxel, Brodersen and coworkers were able to visualize the growth of water droplets inside refilling vessels. Their results appear to confirm both the role of ray parenchyma cells in refilling and the importance of hydraulic isolation of the refilling vessel.

X-ray microtomography, however, is not limited to synchrotron sources: so called ‘desktop’ XMT systems, capable of sub-micron voxel resolution, are becoming increasingly popular. Although a synchrotron offers better temporal resolution and superior beam intensity, desktop scanners are far more easily available to most researchers, and require fairly little user experience to operate efficiently. A home lab –based system also allows conducting long-term studies that could not be carried out within a beam time allocation of a few days. Such systems have already demonstrated their capabilities in visualizing and quantifying xylem anatomy [22,23], but so far there have been few attempts to image the xylem of live trees using these systems. In a larger scale, such a system has already been applied to imaging the development of maize seeds [24], and a similar XMT system to the one used in this study was recently used to observe the growth of *Arabidopsis thaliana*[25]. However, the resolution used with live samples in these studies was not yet sufficient to determine the water contents of individual cells.

In this work, we have investigated the use of a desktop XMT setup in visualizing and quantifying embolism propagation and repair in live saplings and shoot tips of Silver and Curly birch (*Betula pendula* and *B. pendula* var. *carelica*). A significant part of the effort was in devising a suitable sample holder to avoid sample movement artefacts with a live sample, as well as finding suitable scan parameters to minimize radiation exposure to the sample while maintaining a sufficient signal-to-noise ratio to resolve individual xylem cells. The results show that x-ray tube –based XMT equipment can be used to follow the propagation of drought- or freezing-induced embolism and embolism refilling at the cellular level in birch saplings. As simple quantitative metrics of the degree of embolization within the stem, we propose to calculate two values from the cross-sectional tomography images: the number of embolized vessel cells, and the percentage of stem cross-section that consists of embolized xylem. The latter parameter is abbreviated PCS, or percentage of cavitated stem.

## Results

A typical x-ray microtomography scan consists of collecting from a few hundred to several thousands of transmission radiographs while rotating the sample in the x-ray beam. From the set of acquired radiographs, termed *projections*, one can calculate the spatial distribution of the linear attenuation coefficient *μ* within the sample. The end result is the tomographic *reconstruction*, i.e. a digital three dimensional grayscale image, where high gray values correspond to more attenuating material. Each cubical volume element is termed a *voxel* (cf. picture element = > pixel), and the edge length of one element is called the *voxel size* of the reconstruction.

**Figure 1:**
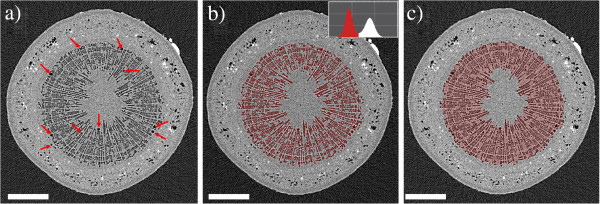
**Quantification of xylem embolism in the stem of a Curly birch sapling. a**) Sap-filled cells and cell walls are shown in light grey. Sap-filled cells and cell walls are shown in light grey, air-filled embolized cells in a dark grey or black. In a wide zone around the pith, the majority of the wood fibers are embolized, even though most vessels remain water-filled. This zone is highlighted in red in **c**). The area of the cavitated zone divided by the area of the whole stem gives the percentage of cavitated stem, or PCS value (37.1%). **b**) Shows an intermediate step in the PCS calculation, along with a grayvalue histogram of the data. Another figure of importance is the number of embolized xylem vessels in the cross section. The arrows in **a**) point to the 10 embolized vessels in this cross-section. Scale bars are 400 μm. The white object on the upper-right edge of the sample is a Blu-Tack marker used to help in locating the same position of the sample.

Figure 1 shows one axial cross section through the 3D reconstruction of a Curly birch stem. By gray value, the image can be divided into three components: air/water vapor in gas-filled cells has a low *μ*, and is seen as dark gray, similar to the air surrounding the sample. The attenuation coefficients of the wood cell wall and sap are higher than that of air, but very close to each other: sap-filled xylem cells as well as the pith and phloem are seen as light-gray areas. However, it is possible to distinguish the boundary between xylem and phloem due to a slight difference in gray value and the presence of air or vapor-filled areas in the phloem (visible as dark spots). The bright heavily attenuating (white) spots that are very abundant in the phloem are mineralized sclereids, or stone cells. The abundance of stone cells was found to be a very useful feature for ‘navigating’ through the reconstruction. Utilizing the stone cells as ‘landmarks’ made it significantly easier to find the same vertical position from scan to scan, despite the cells’ changing water contents and slight alterations to the orientation of the sample.

The calculation of our chosen metrics for the degree of embolization in the stem is also illustrated. Embolized vessels are readily identifiable as larger dark areas within the xylem, and their number can be simply counted in the image. The PCS value is the ratio between the area highlighted with red in Figure 1c, and the total stem cross-sectional area.

### Refilling of embolized xylem due to changes in temperature or lighting conditions

**Figure 2:**
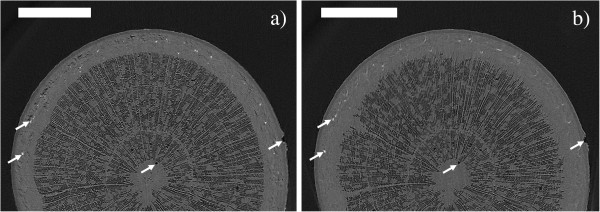
**XMT reconstructions from the stem of a Silver birch sapling.** Axial cross-sections of sample A, uprooted in late October. Image **a**) was taken immediately after bringing the sample indoors, image **b**) after two days in room temperature. In the image **b**), refilling of previously embolized fibers near the edges of the xylem can be seen. Scale bars 800 μm. To guide the eye, the arrows point to the same reference points in the two images.

**Figure 3:**
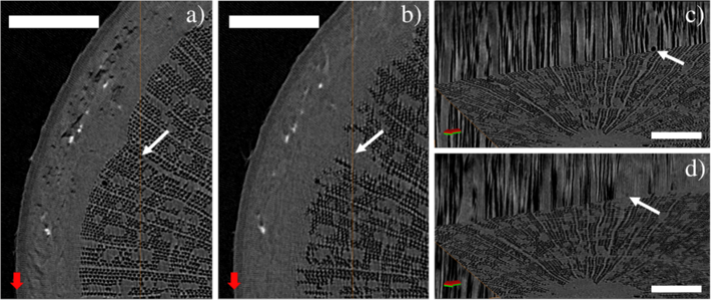
**Refilling of embolized fibers due to increased temperature.** Area near the left edge of Figure 2 shown in greater detail. Images **a**) and **c**) are taken immediately after bringing the sample indoors, **b**) and **d**) after two days at room temperature. Images **a**) and **b**) show axial cross-sections, **c**) and **d**) corresponding views, where also a longitudinal cross-section is shown. In **a**) and **b**), the position of the longitudinal cross section is indicated by the orange vertical line. Scale bars are 350 μm in panels **a**) and **b**), 200 μm in panels **c**) and **d**). In each panel, the white arrow points to the same ray cell, which has been refilled between scans. The red-green compass arrow is intended as a visual aid to understanding the relative orientation of the cross-sections.

We were able to visualize the successful refilling of embolized xylem conduits on two occasions, the first of which is illustrated in Figures 2 and 3. An already leafless sapling of Silver birch (sample A), approximately 40 cm in height, was uprooted in late October and scanned immediately after bringing the sample indoors. In this initial scan, most of the xylem fibers are already embolized due to decreasing temperatures, leaving only a narrow range of sap-filled fibers near the phloem. Most of the vessels, however, were still sap-filled throughout the stem. In the complete cross-section, we counted a total of 11 embolized vessels. After the initial scan, the sample was kept at room temperature for two days before re-scanning. The second scan clearly showed the refilling of some initially gas-filled fibers, starting from the outer edge of the mainly embolized part of the stem. Figure 3 shows axial and longitudinal sections of a smaller area in the two scans, where also the refilling of four initially embolized ray cells can be observed. A radial cross-section of the same region is presented in Additional file 1, showing that these four cells indeed form part of the ray.

**Figure 4:**
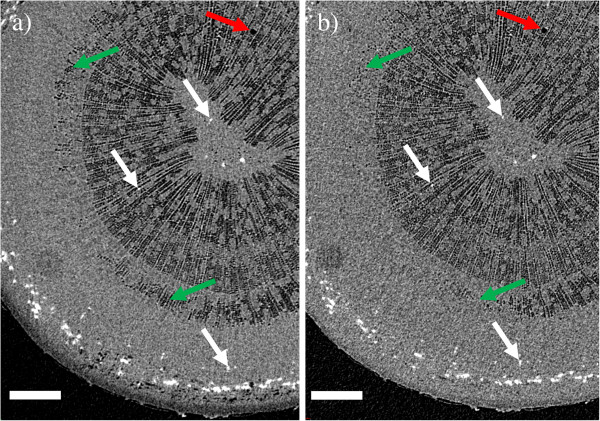
**Refilling of embolized fibers due to changes in lighting conditions.** XMT images showing refilling of embolized wood fibers in the shoot tip of a young Silver birch (sample B). **a**) Image taken immediately after excision in the afternoon, and **b**) the same tissue after maintaining the sample in darkness for one hour. To guide the eye, the white arrows point to the same sclereids in both images. Green arrows indicate areas where refilling of fibers occurred between the scans. Scale bars 350 μm. The single embolized vessel observed in the sample is near the upper-right corner of the images (red arrow).

**Figure 5:**
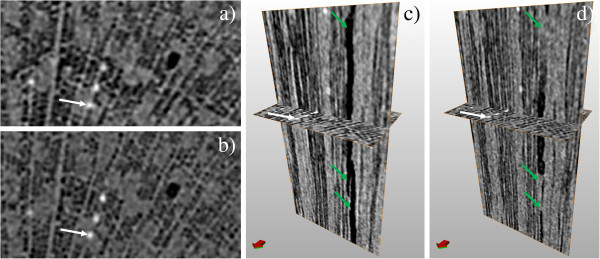
**Partial refilling of a vessel element.** The images show the surroundings of the embolized vessel in Figure 4 in greater detail, with images **a**) and **c**) corresponding to Figure 4a, and **b**) and **d**) corresponding to Figure 4b. The axial cross-section shown in all panels is approximately 300 × 600 μm^2^. **c**) and **d**) also depict a longitudinal section, with green arrows indicating where the vessel was partially refilled between the two scans. As a common point of reference, the white arrow points to the same sclereid in each image.

A similar refilling was also observed in a shoot tip of an approximately 3 m tall Silver birch (sample B) the following summer. The top 40 cm of the main stem was excised on a sunny afternoon in June, and scanned immediately after excision under bright illumination. The lights were then turned off, and the sample scanned again after 1 hour in darkness. The stem was cut under water, and the cut surface kept submerged for the entire experiment. From the results (Figures 4 and 5) we see that most of the fibers were initially embolized near the center of the stem, but sap-filled near the phloem. Only a single vessel was found to be embolized in the scanned section. Maintaining the sample in darkness, the outermost embolized fibers were refilled (Figure 4), and even the embolized vessel started to refill. Figure 5 shows water entering the vessel, both at the ends of a vessel element, as well as in the middle, similar to that previously observed in grapevine [21].

### Embolism propagation in water stressed plants

**Figure 6:**
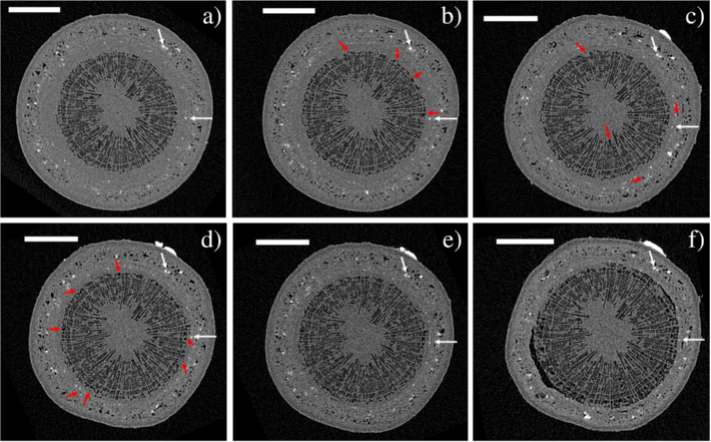
**XMT time series of a Curly birch sapling.** Axial cross-sections of sample C, imaged **a**) in the initial (well-watered) state and **b**) the following day, **c**) 5 days, **d**) 6 days, **e**) 8 days and **f**) 42 days after the initial scan. Watering of the sample was stopped after the initial scan, and resumed 6 days later, after taking image **d**). Scale bars are 500 μm. The white arrows point to the same two sclereids in each image, red arrows indicate xylem conduits which have been embolized since the previous image was taken. The highly attenuating (white) object in the upper-right corner of images c-f is a Blu-Tack marker which was attached to the sample to aid re-positioning the sample for imaging.

**Figure 7:**
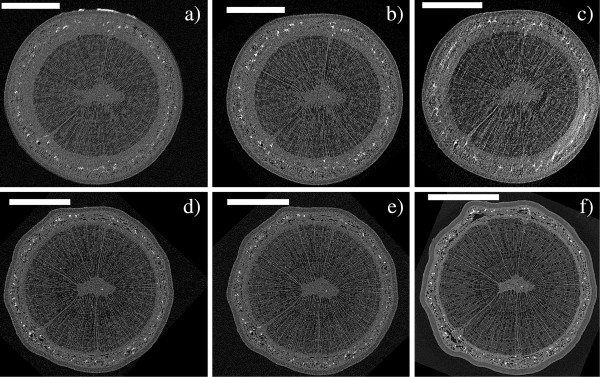
**XMT time series of a Silver birch sapling.** Axial cross-sections of sample D, imaged **a**) in the initial (well-watered) state and **b**) 6 days, **c**) 8 days, **d**) 13 days and **e**) 14 days after the initial scan. Watering of the sample was stopped after the initial scan, and resumed 14 days later, after taking image **d**). Image **f**) shows the same stem cross-section after the sample has been dried in room temperature for 4 months. Scale bars are 1 mm.

**Figure 8:**
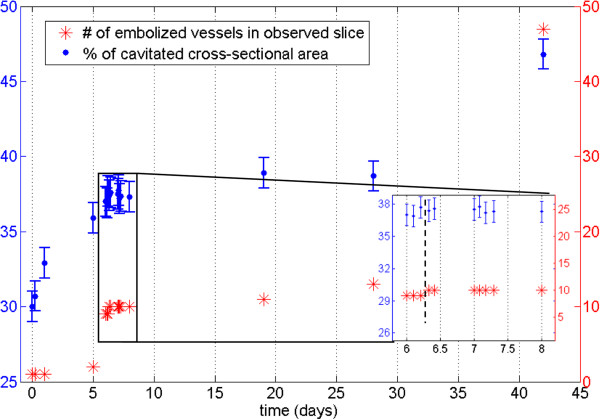
**Quantification of stem embolism in Curly birch.** Percentage of cavitated stem (PCS) values (blue) and the number of embolized vessel cells (red) from a Curly birch sapling (sample C) as a function of time. The inset shows a close-up of data points 5-14, with the vertical dashed line indicating when watering of the sample was resumed.

**Figure 9:**
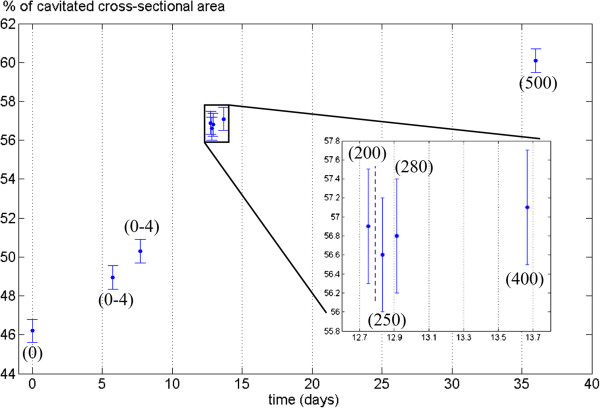
**Quantification of stem embolism in Silver birch.** Percentage of cavitated stem (PCS) values from a Silver birch sapling (sample D) as a function of time. The inset shows a close-up of data points 4-7, with the vertical dashed line indicating when watering of the sample was resumed. The numbers in parentheses are estimates of the number of cavitated vessels in the observed slice. The dried sample (Figure 7f) had a PCS value of 61% and approximately 600 vessels.

We also conducted a long-term experiment, in which watering of live saplings was ceased and embolism formation monitored with repeated XMT scans of the same stem section. The extent of embolization was quantified by calculating the PCS values and counting the number of embolized vessels within a selected cross-section through the sample. Figures 6 and 7 show the observed cross-section of two samples (a Curly birch (C) and a Silver birch (D) sapling) at different stages of the experiment, Figures 8 and 9 the corresponding PCS values and the embolized vessel counts.

Even in the well-watered state, most wood fibers in a large portion of the stem (up to 46%) were found to be embolized. This percentage also started to increase immediately after watering was stopped. However, only isolated vessels were embolized in the initial state, and it was only after several days into the drought that a significant increase in the number of embolized vessels was observed.

Surprisingly, we did not observe reversal of embolisms when watering of the plants was resumed after the drought. After re-watering, however, both the PCS value and the number of embolized vessels were increased considerably slower than during drought conditions. Despite this, our samples did not make a full recovery from the drought, beginning to wilt some weeks after the start of the experiment. Whether this was caused by the ionizing radiation dose, or the prevailing particularly adverse weather conditions, is unknown.

## Discussion

### Applicability of desktop XMT to studying xylem embolism

With the presented results, we have aimed to demonstrate that also desktop x-ray microtomography systems can provide valuable additional information to complement the conventional methods used for xylem embolization studies. Based on the reconstructions, the cavitation of xylem conduits in a water-stressed sapling can be observed and quantified with minimal interference with the sample. Even refilling of embolized conduits can be observed, under favorable conditions.

The XMT system described here is fairly simple to operate even for the inexperienced user, and requires only limited knowledge of the physics or mathematical background of the method. Scan parameter selection is nevertheless a vital phase in the experiment, as e.g. the number of acquired projections, exposure time, and imaging geometry can be modified to produce scans of varying quality. A fast but noisy overview scan can be carried out in less than half an hour, while choosing higher magnification and longer exposure times will improve image resolution, but also increase radiation dose to the sample. When imaging live samples, sample mounting also requires careful consideration, firstly to avoid sample movement artefacts, and secondly to ensure that the sample is not harmed in the mounting if it is to be scanned again at a later time. These two objectives are somewhat conflicting: in order to preserve the sample for further experiments, applying excessive force to the stem should be avoided, while secure mounting would be easiest to achieve by e.g. tightly clamping the sample in place with screws. This emphasizes the need for selecting a suitably sized sample that is tall enough to be fitted into the scanner (with the present setup, the stem must be fairly straight and branchless for the first 15 cm), but not so massive that it would easily shift during the scan, even if held only lightly in place. A smaller sample is also easier to fit in the field-of view (FOV) of the scanner, and the reconstruction is less likely to suffer from undersampling caused by acquiring only a limited number of projections. An example can be seen by comparing images 6 and 7: there is no great difference in nominal resolution between the scans, but image quality in Figure 6 is noticeably better, due to slight movement artefacts and undersampling in reconstructions of the larger Silver birch sample (Figure 7a-7e). Figure 7f (taken after the sample was dried) illustrates the trade-off between scan duration and reconstruction quality: using significantly more exposures and shorter angular sampling resulted in reduced noise, but also in a three- to eight-fold increase in scan duration when compared to images 7a-7e.

### XMT results and hydraulic conductivity

Compared with e.g. hydraulic conductance measurements, the additional information that desktop XMT systems provide is the spatial distribution of embolized conduits within the stem: from a PLC measurement, one only gets a number representing the percentage of hydraulic conductance lost to embolism. On the other hand, an XMT scan is essentially an image of the stem, allowing us to determine not only the quantity of embolisms, but also the types of embolized cells, and where they are located within the stem. Moreover, the XMT measurement is non-destructive, so it should be possible to make both measurements with the same samples. Combining in vivo XMT scans of live saplings with PLC and/or water potential measurements should therefore be of great interest and give a more detailed understanding of what exactly happens in the stem as conduits are embolized and refilled.

Although the focus in this work was in evaluating the capabilities of desktop x-ray systems for scanning live trees, we can already point out some interesting observations from the results. In particular, it appears that drought conditions result in a rise of the PCS value (i.e. embolization of wood fibers and reduction in phloem area) before the vessels start to embolize. One should especially consider that the PCS value in this case reflects the cross-sectional area of embolized fibers versus the total stem cross-section. The values reported in Figures 8 and 9 should therefore be compared with those calculated from the same stem sections, after being substantially or completely dried (Figures 6f and 7f, PCS values 46% and 61%). This finding is quite surprising, since water transport in angiosperms is believed to occur mainly through the vessels: since vessels are also larger, one would expect them to be more vulnerable to cavitation than the fibers. Yet drought conditions are first manifested as embolisms in the less conducting cell type. We could hypothesize that this is due to the capacitive effect of wood fibers (see e.g. [26]) and the phloem; these tissues act as an internal source of water to the sapling, supplying the conducting vessels with water via lateral transport, while having only a minor contribution to the axial water transport capacity. Phloem may also be acting as a source for water refilling the xylem conduits between scans (e.g. [7]), which could account for the observed reduction in phloem area. Vessels are embolized only after this internal water reservoir is depleted in prolonged drought conditions. Similar hydraulic capacitance effects within the stem have recently been discussed elsewhere [27,28]. As noted, though, the main objective in this study was simply to establish whether the desktop XMT method can be used for water transport studies; detailed interpretation of the results would require both a larger series of samples and combining the XMT data with conductance and water potential measurements.

### Comparisons with other imaging methods

Compared with a synchrotron x-ray tomography beamline, a desktop XMT setup has both advantages and disadvantages with respect to water dynamics studies in trees. The main technical drawback when using an x-ray tube is the lower photon flux, which results in longer scan times and worse signal-to-noise ratio than that available at a synchrotron source. At a synchrotron source, also the radiation dose to the sample is reduced, since a monochromatic x-ray beam can be used for imaging. The spatial resolution achievable with both methods is comparable, and while the images obtained with a desktop setup are somewhat noisier, the image quality is still sufficient to determine the water contents of individual conduits. By optimizing the scan parameters, the scan times could be reduced to approximately 0.5 hours per scan (e.g. scan in Figure 1). Since large changes in xylem hydraulic conductance and leaf water potential have been observed to take place in hours rather than minutes [10,17,21], the method has potential applications in embolism and refilling studies despite the rather poor temporal resolution. The greatest advantage desktop XMT has over its synchrotron-based alternative is availability: there are perhaps twenty synchrotron imaging beamlines in the world, and access to them is highly competed for. The application process for a beamtime allocation of a few days must usually be started several months prior to the experiment planned. Desktop XMT systems, on the other hand, are getting increasingly popular at universities and other research institutes around the world, and are relatively easily accessible. Construction of a custom-built scanner specifically suited for scanning live plants is possible even for an individual institution.

While the resolution of all tomographic methods (both synchrotron and home-lab based) is somewhat poorer than what can be achieved with 2D-microscopy (optical or SEM), XMT also has its advantages compared with these methods. The first of these is that a live sample can be scanned with minimal preparation, eliminating the laborious sectioning and freezing or staining processes. The only challenge in this respect is mounting the sample securely to avoid sample movement artefacts in the reconstruction. XMT data is also three-dimensional, which means there is no need to make a separate experiment to obtain images in different directions. Instead, the XMT reconstruction can be digitally ‘sectioned’ in arbitrary directions, or even viewed as a three dimensional rendering. When investigating samples in vivo, using XMT also avoids any potential artefacts relating to physically cutting or the sample or vitrifying the tissue with liquid nitrogen.

### Radiation dose considerations

Perhaps the biggest drawback with using desktop XMT systems for long-term studies in vivo is that the scanned stem section is exposed to a considerable dose of ionizing radiation (estimated surface dose is up to 100 kGy): this cannot be ruled out as a potential reason for the observed wilting of samples C and D some weeks after the initial XMT scan. However, due to the small size of the x-ray source, e.g. the leaves of the sample are exposed to less than 1 Gy during a one-hour scan, which is well below the observed threshold for acute radiation effects [29]. It should also be noted that the effects of ionizing radiation are primarily manifested in tissues undergoing cell division [30]. In the stem, the only still dividing tissue is the cambium; other tissues are mostly composed of cells that are either dead (xylem vessels and fibers), or alive but no longer dividing (phloem cells and xylem parenchyma). An alternative explanation for the wilting is the unfortunate timing of the experiment: these samples were scanned mostly in July 2010, which by Finnish standards was unprecedentedly warm: according to weather data from the SMEAR III station located on-campus, even the 25^th^ percentile of daily temperatures in July 2010 (18.6°C) was higher than the 30-year average mean (17.2°C)^a^. As the samples were kept in pots outdoors between scans, it is possible that the exceptionally warm weather played a major role, preventing the recovery of the samples from the induced water stress.

## Conclusions

This study illustrates the capabilities of a desktop XMT system in studying xylem embolism in living plants. The method is useful for visualizing the water status of a young sapling in vivo, at the level of individual cells. Refilling of embolized conduits can be observed as well, at least when induced by root pressure. As compared to traditional methods of measuring water status, direct imaging of the vessel (or fiber) contents is very reliable, reducing the risk of experimental artefacts or misinterpretation of the raw data. Since XMT scanning is non-destructive, it can be used easily to complement other techniques, and combining leaf water potential or PLC measurements with in vivo information of the water content of the cells could provide new insight into the dynamics of sap ascent and embolism propagation in tree stems. The main advantages of home-lab XMT systems over synchrotron beamlines are easier availability especially for long sample series and the need for relatively little experience for efficient use. However, longer scan times and greater radiation dose to the sample may be problematic especially if tissues with dividing cells are imaged.

## Methods

### XMT system

**Figure 10:**
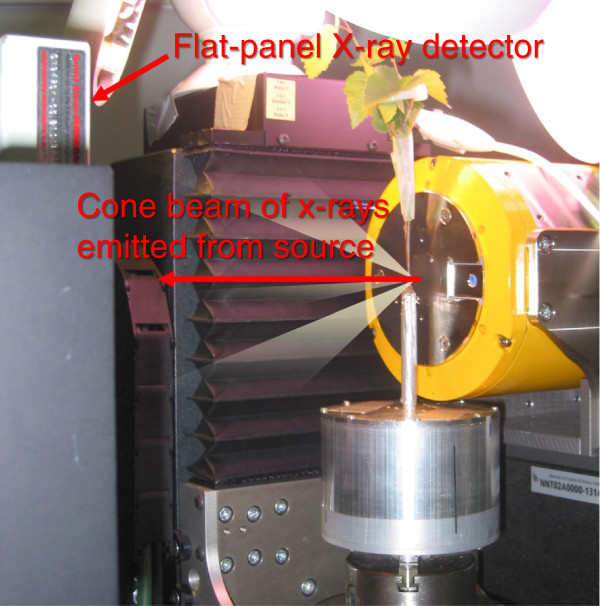
**The XMT setup.** X-rays are emitted in a wide cone beam from the x-ray tube on the right, and detected with the flat-panel detector to the left. Only the upper corner of the detector is visible from behind its mounting.

The XMT scans were conducted using microtomography equipment (Nanotom 180 NF) by Phoenix|x-ray Systems and Services (Wunstorf, Germany; currently owned by GE Measurement and Control Solutions). The equipment, shown in Figure 10, consists of a nanofocus x-ray source with a tungsten target, a high-resolution computer-controlled sample positioning and rotation stage, and a CMOS flat panel x-ray detector (Hamamatsu Photonics, Japan) with 2304 × 2304 pixels, each 50 μm × 50 μm in size.

In our setup, one XMT scan consists of up to a few thousand projections, taken over a full 360° rotation of the sample. To reduce noise, each projection image is acquired as the average of several exposures. Reconstructions are performed with datos|x -reconstruction software, provided by the XMT equipment manufacturer. The software uses a cone-beam filtered back-projection based on the FDK algorithm [31].

### Samples

We present results obtained from four different samples: initial scans were performed on a wild-grown intact sapling of Silver birch (*Betula pendula* Roth, sample A) from Hausjärvi in southern Finland, and a shoot tip of Silver birch growing in Kumpula, Helsinki (sample B). A more systematic scan series was taken of commercially grown pot plants, one Curly birch (*Betula pendula* var. *carelica* (Merckl.) Hämet-Ahti, sample C) and one Silver birch (sample D) sapling provided by Forestum Oy (Helsinki, Finland). Sample A was scanned in late October, and samples B-D in June-July the following year.

The sample holder used in this study is shown in Figure 10. It consists of an aluminum pot (diameter 9 cm, depth 6.5 cm) attached to the manipulator stage, a two-piece lid, and an aluminum collar (diameter 4 mm) to support the bottom part of the stem. The scanned section was approximately 8 cm above the soil surface, just above the top of the collar. The stem was secured in place inside the collar using beeswax. When scanning sample B, the pot was filled with water. To fit the intact Silver birch samples (A and C) into the pot, it was necessary to cut off the bottom 2 cm of their roots. With samples B-D, also the lighting conditions were varied during the experiment: daylight was simulated by illuminating the samples with three 15 W plant lights (Megaman W1514P), and night by scanning the samples in darkness.

### Selection of scan parameters

Setting up an XMT scan can be viewed as an optimization problem between voxel size, noise level, scan duration, and radiation dose to the sample. In this work, the magnification, exposure time and number of projections were varied in order to find the best configuration for imaging live plants, i.e. the shortest scan time that still results in reconstructions of acceptable quality. We will summarize the key points to be considered when selecting these parameters as they apply to the system used in this study. For a comprehensive discussion, the reader is referred to e.g. the introductory text by S.R. Stock [32].

The voxel size of the reconstruction is proportional to the ratio of the distances from the x-ray source to the sample and the detector. This implies a trade-off between magnification and required scan time or radiation dose induced to the sample: magnification can be increased either by moving the sample closer to the source where also the radiation dose rate is higher, or by moving the detector farther from the source, requiring longer exposures to collect the same amount of x-rays. However, the voxel size alone does not determine the scan resolution: the size of the details that can actually be resolved in a reconstruction is also dependent on the signal-to-noise ratio and the presence or absence of various artefacts, with sample movement and undersampling being perhaps the most relevant to imaging live plants.

The signal-to-noise ratio can be improved by increasing the x-ray tube current, by increasing the exposure time, or by averaging over more exposures to obtain each projection. Undersampling effects in the reconstruction can be reduced by acquiring more projections, again at the cost of increased scan duration. However, since some undersampling is inevitable, the required number is application-dependent: there are sufficient projections when the required analysis can be carried out based on the reconstruction.

Contrast in the transmission images can be optimized by selecting the correct acceleration voltage of the x-ray tube. As a general rule, heavy elements absorb x-rays more easily than light elements, and low-energy x-rays get absorbed more easily than high-energy x-rays. Because a tree is mostly composed of light elements, the acceleration voltage was held at 50-70 kV, which produces a maximum in the beam spectrum at photon energies of approximately 20-40 keV. This represents a compromise between the elemental composition of the sample, which would have warranted an even lower voltage, and the characteristics of the detector, which is inefficient at low photon energies. Choosing the lowest possible acceleration voltage also has the effect of reducing the radiation dose to the sample. The current was selected between 150-300 μA to maximize intensity without sacrificing the detection of details by having to enlarge the source size. To obtain the best possible resolution, the samples were placed as close as possible (10-20 mm) and the detector at 200-250 mm from the x-ray tube window. This resulted in voxel sizes ranging from 1 × 1 × 1 μm^3^ to 2.5 × 2.5 × 2.5 μm^3^.

It is preferable that the projections are not truncated horizontally, i.e. the sample is small enough for some length of the stem to fit entirely in the FOV. The relatively thick stems of Silver birch samples (A, B and D) could be visualized by either moving the sample away from the x-ray tube (sacrificing resolution), or using the so-called ‘virtual detector’ mode, in which the projections at each angle are acquired using two different horizontal positions of the detector. The two images are then patched together into one large image, effectively doubling the FOV.

The scans from sample A were made mainly to explore the applicability of the sample setup for imaging live samples. The scans were therefore quite long (9 h), with a voxel size of 1.2 μm.

The sample was uprooted and scanned intact in late October, after shedding its leaves. The first scan was done the following day, keeping the sample outdoors in the meantime (with low temperatures slightly below 0°C). After the first scan (starting at 10:00, with outside temperature >0°C), the sample was kept in the sample holder at room temperature for two days and scanned again. The number of projections was 1800 in both scans.

Sample B was monitored using shorter (1.5 h) scans with slightly larger voxel size, ~2 μm. The sample was excised from an approximately 3 m tall tree on a sunny afternoon in the summer, when transpiration was highest. To avoid air-seeding of embolisms, the excision was done under water, and the cut end was kept submerged for the duration of the experiment. The first scan was taken immediately after excision, with the sample illuminated by plant lights. The lights were then switched off, and the sample scanned again after one hour in darkness. The number of projections was 1440.

Samples C and D were imaged with still shorter scan times than sample B, while maintaining the voxel size at 2.0-2.5 μm. Both samples C and D were first imaged in the initial (well-watered) state, after which watering was stopped and the water status of the plants monitored by regular scans. Once significant numbers of embolized vessels were observed, watering was resumed, and the plant scanned again after one to a few hours. Follow-up scans were taken at intervals of one to a few days. Sample C was mostly imaged with 60-72 min scans, not utilizing the virtual detector mode, and gradually reducing the number of projections from 1440 to 600. The final four out of 17 scans were made with 600 projections and a total scan time of 30 min. The first 2 scans of sample D were taken in the virtual detector mode, which resulted in scan times of 68-80 min with 1200 projections. For further scans it was possible to fit the sample inside the FOV by using only one detector position, so the scan time could be reduced to 30 min, using 600 projections. After the experiment, a final scan of sample D was made after drying a cut-off section of the stem in room temperature for four months. The final scan (Figure 7f) consisted of 1440 projections, with scan time of 252 min.

### Data analysis

Data analysis is perhaps the most time-consuming phase in an XMT experiment, and certainly the one that requires the most user effort; this is especially true if the data is to be quantified in some fashion, and not simply being used to visually support other measurements. For many tasks, the image processing tools used are very similar to their counterparts used in 2D image processing (see e.g. [33] for an introduction), but the sheer amount of data produced can be a challenge with the file size for one reconstruction easily reaching several gigabytes. Due to the large data size, the figures in this article are somewhat downsampled from those used in the analysis: the original-size cross-sections from which the figures are composed are provided as Additional files 2, 3, 4, 5, 6, 7, 8, 9, 10, 11, 12, 13, 14, 15, 16, 17, 18, 19, 20, 21, 22, 23, 24. All data analyses and visualizations in this work were performed using either Avizo Fire Edition (v. 6.1 & 7.0, VSG technologies, France) or VGStudioMAX (v. 1.3, Volume Graphics, Germany) software.

We used two methods of quantifying xylem embolism from XMT data of a tree stem: calculating the number of embolized vessel cells and the percentage of stem area that consists of cavitated xylem (PCS). Both characteristics were calculated at a single specified cross sectional slice, which could be identified in the reconstructions by using the sclereids as landmarks.

Once the specified slice was found in each reconstruction of the same sample, counting the number of embolized vessels in that slice was trivial. It should be noted that while the noise sometimes made it difficult to identify these from a single cross-sectional image, the fact that the data is 3D proved a great help: an embolized vessel was easy to distinguish from a noise-related cluster of dark voxels, because an embolized vessel was present in several consecutive axial slices. 3D data could also be viewed longitudinally (or at any arbitrary angle) to aid in the identification.

The PCS value calculation requires more complicated image processing. As a first step, all data sets of the sample were resampled to the same voxel size and filtered to remove noise. For the noise removal, bilateral filtering similar to [34] was used, as it is a relatively fast but edge-preserving smoothing method. The effect of the bilateral filtering on the data in Figure 1 is presented in Additional file 25. When applied to birch stem data, bilateral filtering tended to produce bimodal histograms, with the peak at low grayvalues corresponding to lumina of gas-filled cells and the air around the sample, and the peak at higher grayvalues corresponding to sap-filled tissue and the cell walls of gas-filled conduits. After bilateral filtering the PCS calculation was carried out with the following sequence:

1. The data was cropped to 200 slices surrounding the selected slice

2. All connected voxels belonging to the low grayvalue peak were selected using a volume propagation tool (‘magic wand’), starting from a randomly chosen embolized xylem cell.

3. As long as embolized cells clearly belonging to the xylem were left unselected, the volume propagation was repeated starting from one of those cells. The outcome of step 3 is presented in Figure 1b.

4. With noiseless and artefact-free data the selection in Figure 1b could be used as the cross-sectional area of cavitated xylem. However, with significant noise and sample movement artefacts, this proved to be very sensitive to the selected threshold of the volume propagation and input parameters of the bilateral filter. To make the quantification more robust, a 3D morphological closing operation (for a definition in 2D, see ref. [33], pp.627-639) was applied. This has the effect of filling gaps between selected voxels, without expanding the perimeter of the selection.

5. As long as there was more than one set of connected and unselected voxels within the selection, step 4 was re-done, increasing the size of the structuring element by one voxel. The outcome of this step is the selection in Figure 1c.

6. The PCS value was calculated as the area of the last selection divided by the total stem cross-sectional area (obtained by selecting all voxels in the high-grayvalue peak and filling the volume). Lower and upper error bounds for the PCS value were obtained by using in step 5 structuring elements that were smaller and larger by one voxel.

## Endnotes

^a^SMEAR III data courtesy of Dr. Leena Järvi, Division of Atmospheric Sciences, University of Helsinki.

## Abbreviations

PLC: Percentage Loss of hydraulic Conductivity; SEM: Scanning Electron Microscopy; MRI: Magnetic Resonance Imaging; XMT, μCT: X-ray MicroTomography; PCS: Percentage of Cavitated Stem area; FOV: Field-Of-View

## Competing interests

The authors declare they have no competing financial or non-financial interests.

## Authors’ contributions

J-PS and MP designed the experimental procedures used for imaging and the sample mounting, J-PS, KF and RS designed and planned the in vivo experiments. The experiment was carried out and the manuscript drafted by J-PS, with all authors providing comments on the manuscript and the interpretation of the results. All authors have read and approved the manuscript for publication.

## Supplementary Material

Additional file 1 **The figure shows a radial cross-section through the same reconstruction as Figures** 2**a,** 3**a and** 3**c.** The embolized ray cells are indicated by the white arrow. They are surrounded by the still sap-filled cells of the same ray, which are visible in light gray. Scale bar is 350 μm. Click here for file

Additional file 2 **The original size bilateral filtered data of Figure** 1 **as a tif image.** The data is grayscale in unsigned 16-bit integer format. Data is resampled to a pixel size of 2.50 × 2.50 μm^2^, when the original voxel size in the reconstruction was 2.05 × 2.05 × 2,05 μm^3^. Click here for file

Additional file 3 **The original size bilateral filtered data of Figures** 2**a and** 3**a as a tif image.** The data is grayscale in unsigned 16-bit integer format. Pixel size is 1.18 × 1.18 μm^2^. Click here for file

Additional file 4 **The original size bilateral filtered data of Figures** 2**b and** 3**b as a tif image.** The data is grayscale in unsigned 16-bit integer format. Pixel size is 1.12 × 1.12 μm^2^. Click here for file

Additional file 5 **The original size bilateral filtered data of the vertical cross-section in Figure** 3**c.** The data is grayscale in unsigned 16-bit integer format. Pixel size is 1.18 × 1.18 μm^2^. Click here for file

Additional file 6 **The original size bilateral filtered data of the vertical cross-section in Figure** 3**d.** The data is grayscale in unsigned 16-bit integer format. Pixel size is 1.12 × 1.12 μm^2^. Click here for file

Additional file 7 **The original size bilateral filtered data of the cross-section in Figure** 4**a.** The data is grayscale in unsigned 16-bit integer format. Pixel size is 2.00 × 2.00 μm^2^. Click here for file

Additional file 8 **The original size bilateral filtered data of the cross-section in Figure** 4**b.** The data is grayscale in unsigned 16-bit integer format. Pixel size is 2.00 × 2.00 μm^2^. Click here for file

Additional file 9 **The original size bilateral filtered data of the cross-section in Figure** 5**a.** The data is grayscale in unsigned 16-bit integer format. Pixel size is 2.00 × 2.00 μm^2^. Click here for file

Additional file 10 **The original size bilateral filtered data of the cross-section in Figure** 5**b.** The data is grayscale in unsigned 16-bit integer format. Pixel size is 2.00 × 2.00 μm^2^. Click here for file

Additional file 11 **The original size bilateral filtered data of the vertical cross-section in Figure** 5**c.** The data is grayscale in unsigned 16-bit integer format. Pixel size is 2.00 × 2.00 μm^2^. Click here for file

Additional file 12 **The original size bilateral filtered data of the vertical cross-section in Figure** 5**d.** The data is grayscale in unsigned 16-bit integer format. Pixel size is 2.00 × 2.00 μm^2^. Click here for file

Additional file 13 **The original size bilateral filtered data of the cross-section in Figure** 6**a.** The data is grayscale in unsigned 16-bit integer format. Pixel size is 2.50 × 2.50 μm^2^. Click here for file

Additional file 14 **The original size bilateral filtered data of the cross-section in Figure** 6**b.** The data is grayscale in unsigned 16-bit integer format. Data is resampled to a pixel size of 2.50 × 2.50 μm^2^, when the original voxel size in the reconstruction was 2.34 × 2.34 × 2.34 μm^3^. Click here for file

Additional file 15 **The original size bilateral filtered data of the cross-section in Figure** 6**c.** The data is grayscale in unsigned 16-bit integer format. Data is resampled to a pixel size of 2.50 × 2.50 μm^2^, when the original voxel size in the reconstruction was 2.05 × 2.05 × 2.05 μm^3^. Click here for file

Additional file 16 **The original size bilateral filtered data of the cross-section in Figure** 6**d.** The data is grayscale in unsigned 16-bit integer format. Data is resampled to a pixel size of 2.50 × 2.50 μm^2^, when the original voxel size in the reconstruction was 2.27 × 2.27 × 2.27 μm^3^. Click here for file

Additional file 17 **The original size bilateral filtered data of the cross-section in Figure** 6**e.** The data is grayscale in unsigned 16-bit integer format. Data is resampled to a pixel size of 2.50 × 2.50 μm^2^, when the original voxel size in the reconstruction was 2.10 × 2.10 × 2.10 μm^3^. Click here for file

Additional file 18 **The original size bilateral filtered data of the cross-section in Figure** 6**f.** The data is grayscale in unsigned 16-bit integer format. Data is resampled to a pixel size of 2.50 × 2.50 μm^2^, when the original voxel size in the reconstruction was 2.10 × 2.10 × 2.10 μm^3^. Click here for file

Additional file 19 **The original size bilateral filtered data of the cross-section in Figure** 7**a.** The data is grayscale in unsigned 16-bit integer format. Data is resampled to a pixel size of 2.30 × 2.30 μm^2^, when the original voxel size in the reconstruction was 2.23 × 2.23 × 2.23 μm^3^. Click here for file

Additional file 20 **The original size bilateral filtered data of the cross-section in Figure** 7**b.** The data is grayscale in unsigned 16-bit integer format. Data is resampled to a pixel size of 2.30 × 2.30 μm^2^, when the original voxel size in the reconstruction was 1.98 × 1.98 × 1.98 μm^3^. Click here for file

Additional file 21 **The original size bilateral filtered data of the cross-section in Figure** 7**c.** The data is grayscale in unsigned 16-bit integer format. Data is resampled to a pixel size of 2.30 × 2.30 μm^2^, when the original voxel size in the reconstruction was 2.26 × 2.26 × 2.26 μm^3^. Click here for file

Additional file 22 **The original size bilateral filtered data of the cross-section in Figure** 7**d.** The data is grayscale in unsigned 16-bit integer format. Data is resampled to a pixel size of 2.30 × 2.30 μm^2^, when the original voxel size in the reconstruction was 2.06 × 2.06 × 2.06 μm^3^. Click here for file

Additional file 23 **The original size bilateral filtered data of the cross-section in Figure** 7**e.** The data is grayscale in unsigned 16-bit integer format. Data is resampled to a pixel size of 2.30 × 2.30 μm^2^, when the original voxel size in the reconstruction was 2.14 × 2.14 × 2.14 μm^3^. Click here for file

Additional file 24 **The original size bilateral filtered data of the cross-section in Figure** 7**f.** The data is grayscale in unsigned 16-bit integer format. Data is resampled to a pixel size of 2.30 × 2.30 μm^2^, when the original voxel size in the reconstruction was 1.28 × 1.28 × 1.28 μm^3^. Click here for file

Additional file 25 **Left: cross-section of the original XMT data used to produce Figure** 1**.** Right: the same data after bilateral filtering to reduce noise (same as Figure 1a). Scale bars are 400 μm. Histograms of the voxel values are also shown; brightness and contrast of the images are set to show a grayvalue of 10200 as black and a grayvalue of 19000 as white. Click here for file
