# Supplementary figures and images for: Visualizing water-filled versus embolized status of xylem conduits by desktop x-ray microtomography
Source: Plant Methods. 2013 Apr 8;9:11. doi: 10.1186/1746-4811-9-11 (PMC3716634; doi:10.1186/1746-4811-9-11)

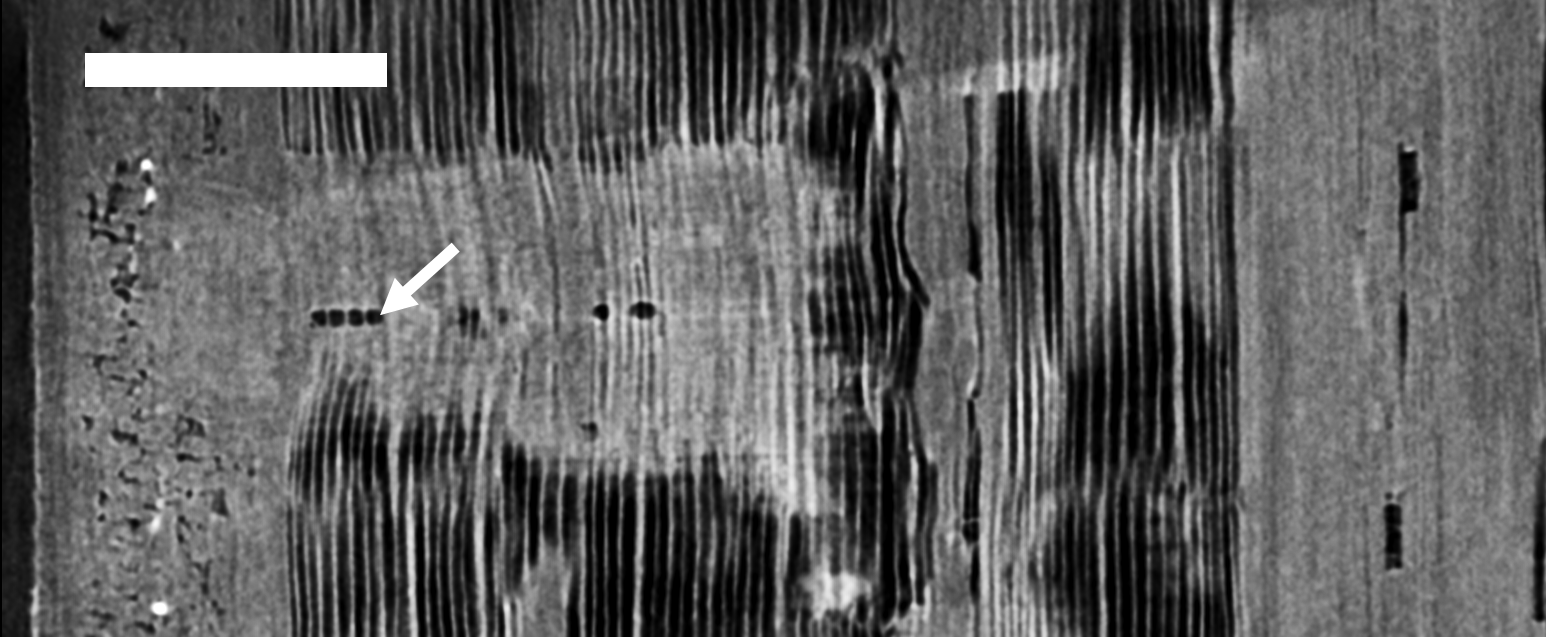

Supplement: Additional file 1 — The figure shows a radial cross-section through the same reconstruction as Figures 2a, 3a and 3c. The embolized ray cells are indicated by the white arrow. They are surrounded by the still sap-filled cells of the same ray, which are visible in light gray. Scale bar is 350 μm. [file 1746-4811-9-11-S1.png]

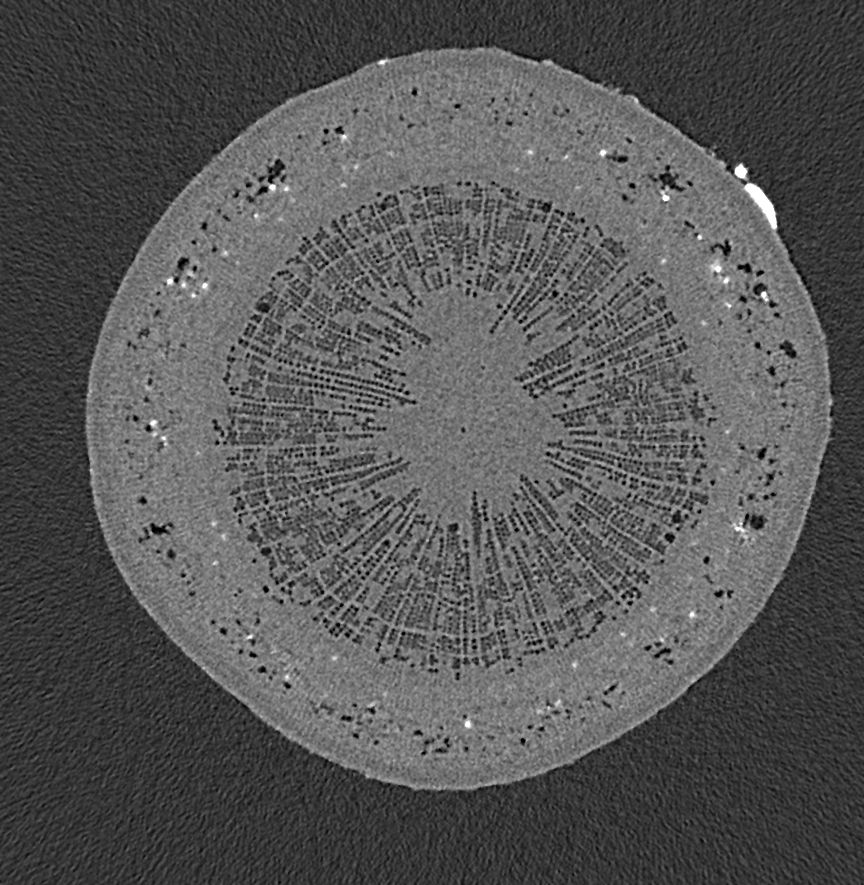

Supplement: Additional file 2 — The original size bilateral filtered data of Figure 1 as a tif image. The data is grayscale in unsigned 16-bit integer format. Data is resampled to a pixel size of 2.50 × 2.50 μm2, when the original voxel size in the reconstruction was 2.05 × 2.05 × 2,05 μm3. [file 1746-4811-9-11-S2.tiff]

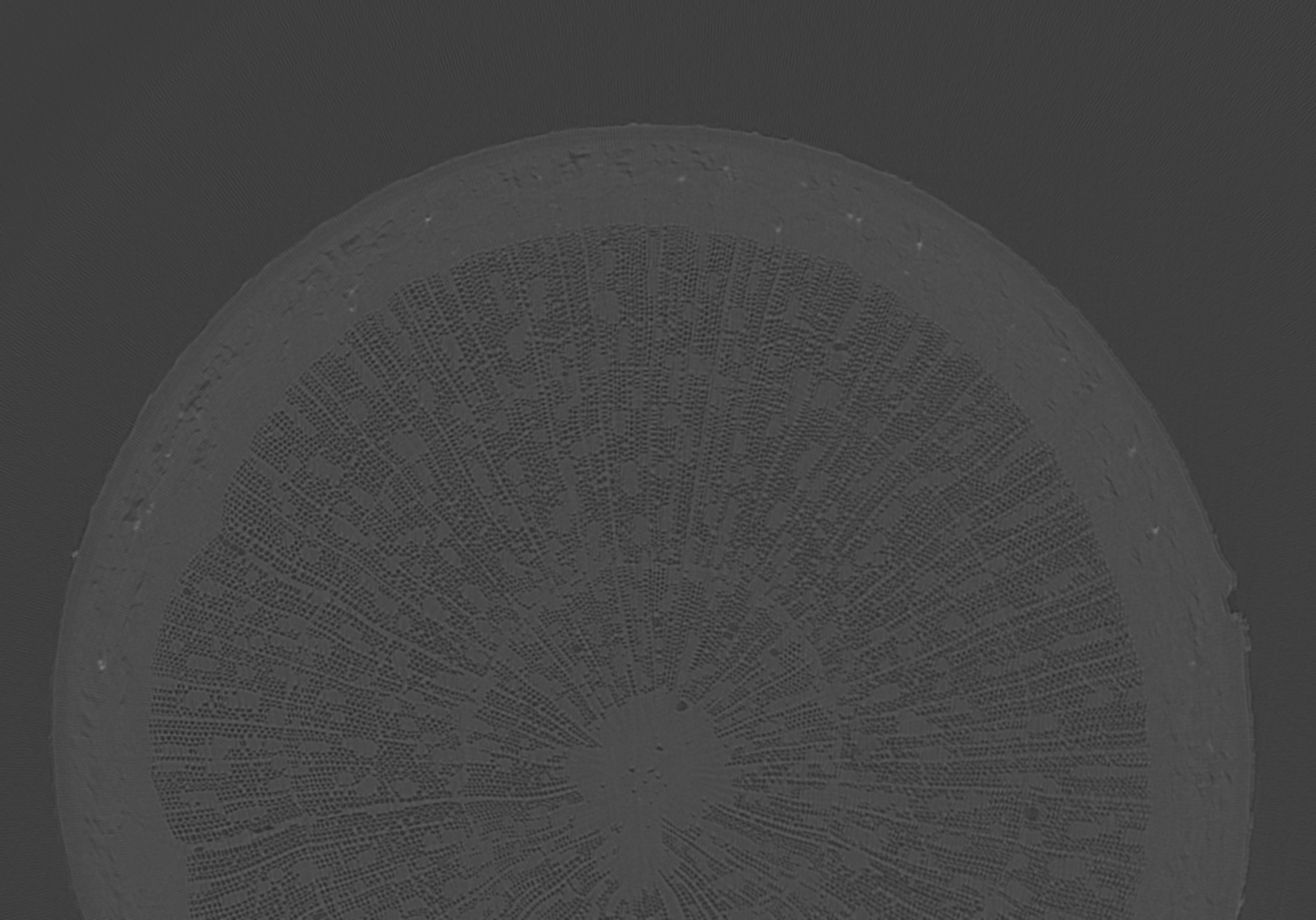

Supplement: Additional file 3 — The original size bilateral filtered data of Figures 2a and 3a as a tif image. The data is grayscale in unsigned 16-bit integer format. Pixel size is 1.18 × 1.18 μm2. [file 1746-4811-9-11-S3.tiff]

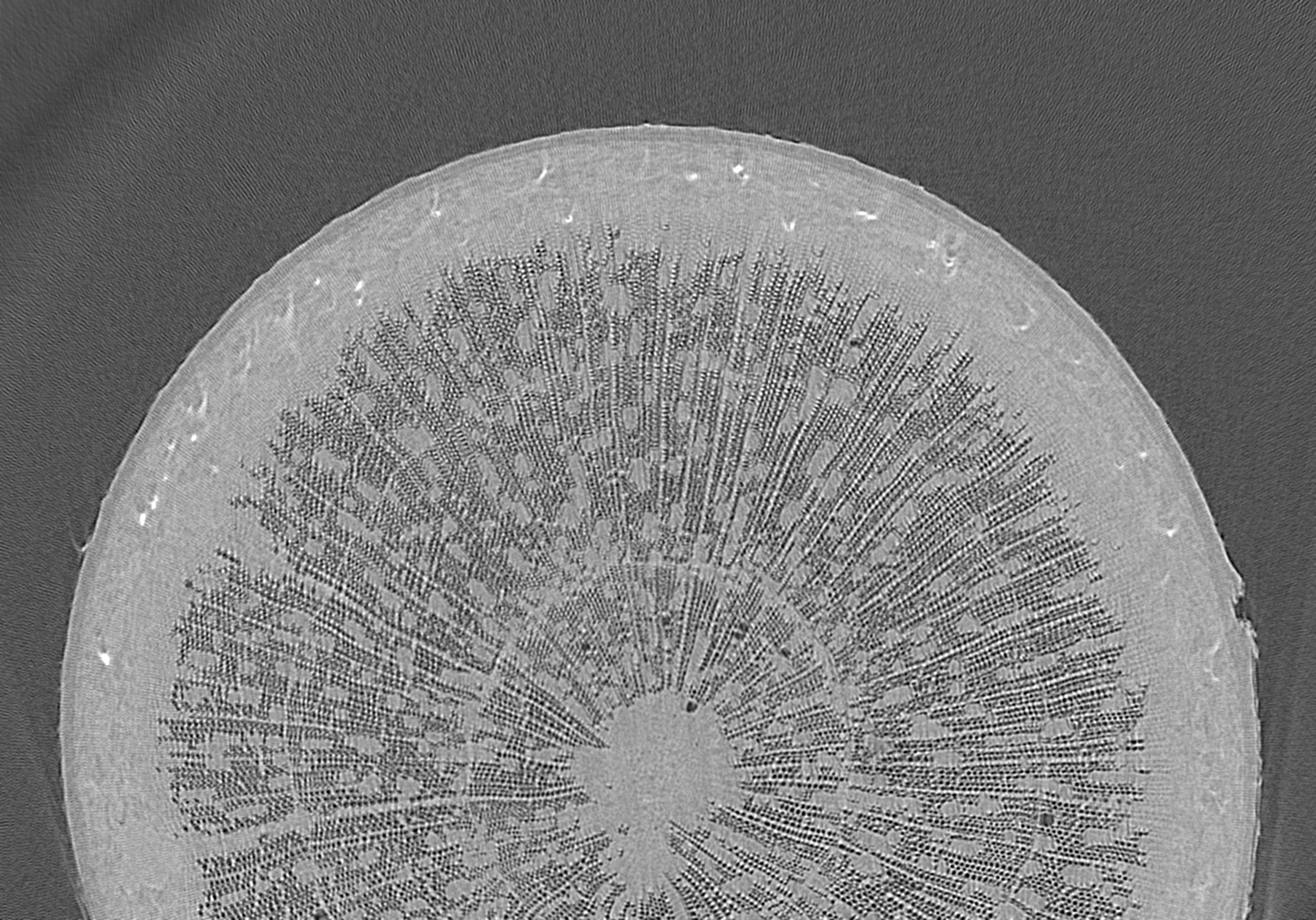

Supplement: Additional file 4 — The original size bilateral filtered data of Figures 2b and 3b as a tif image. The data is grayscale in unsigned 16-bit integer format. Pixel size is 1.12 × 1.12 μm2. [file 1746-4811-9-11-S4.tiff]

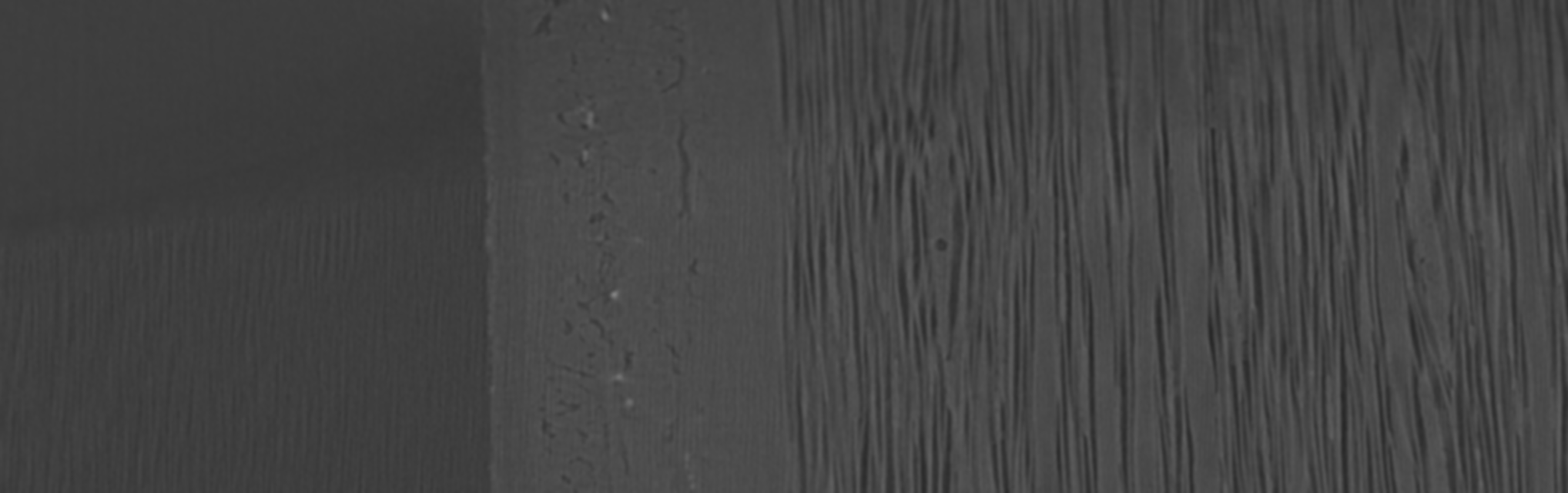

Supplement: Additional file 5 — The original size bilateral filtered data of the vertical cross-section in Figure 3c. The data is grayscale in unsigned 16-bit integer format. Pixel size is 1.18 × 1.18 μm2. [file 1746-4811-9-11-S5.tiff]

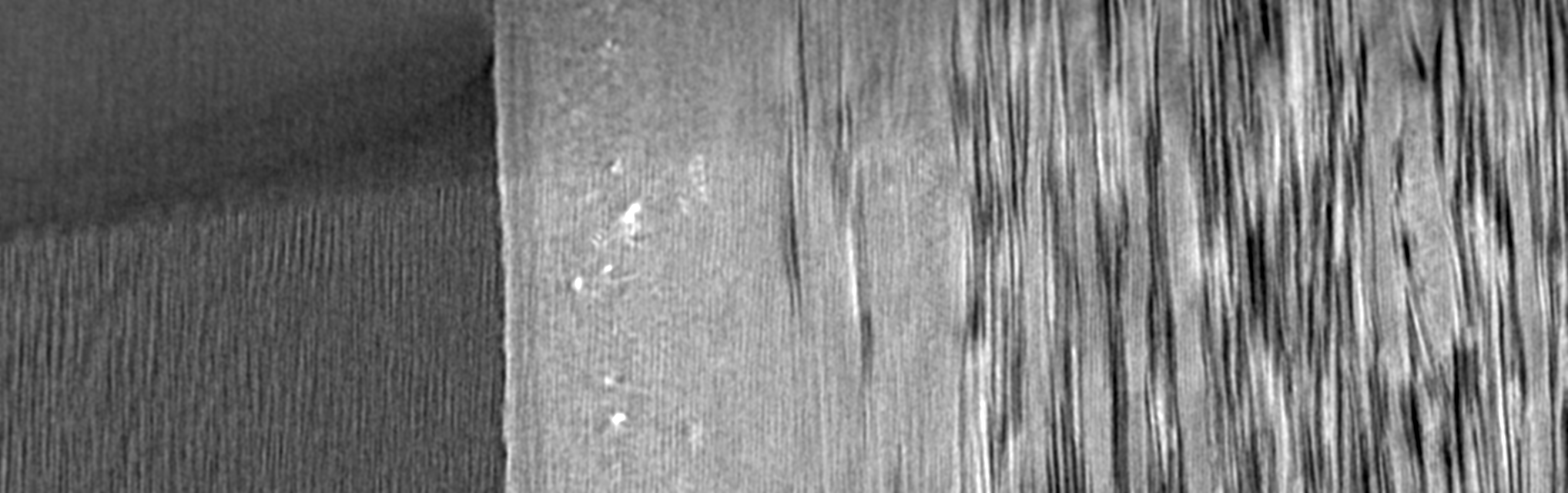

Supplement: Additional file 6 — The original size bilateral filtered data of the vertical cross-section in Figure 3d. The data is grayscale in unsigned 16-bit integer format. Pixel size is 1.12 × 1.12 μm2. [file 1746-4811-9-11-S6.tiff]

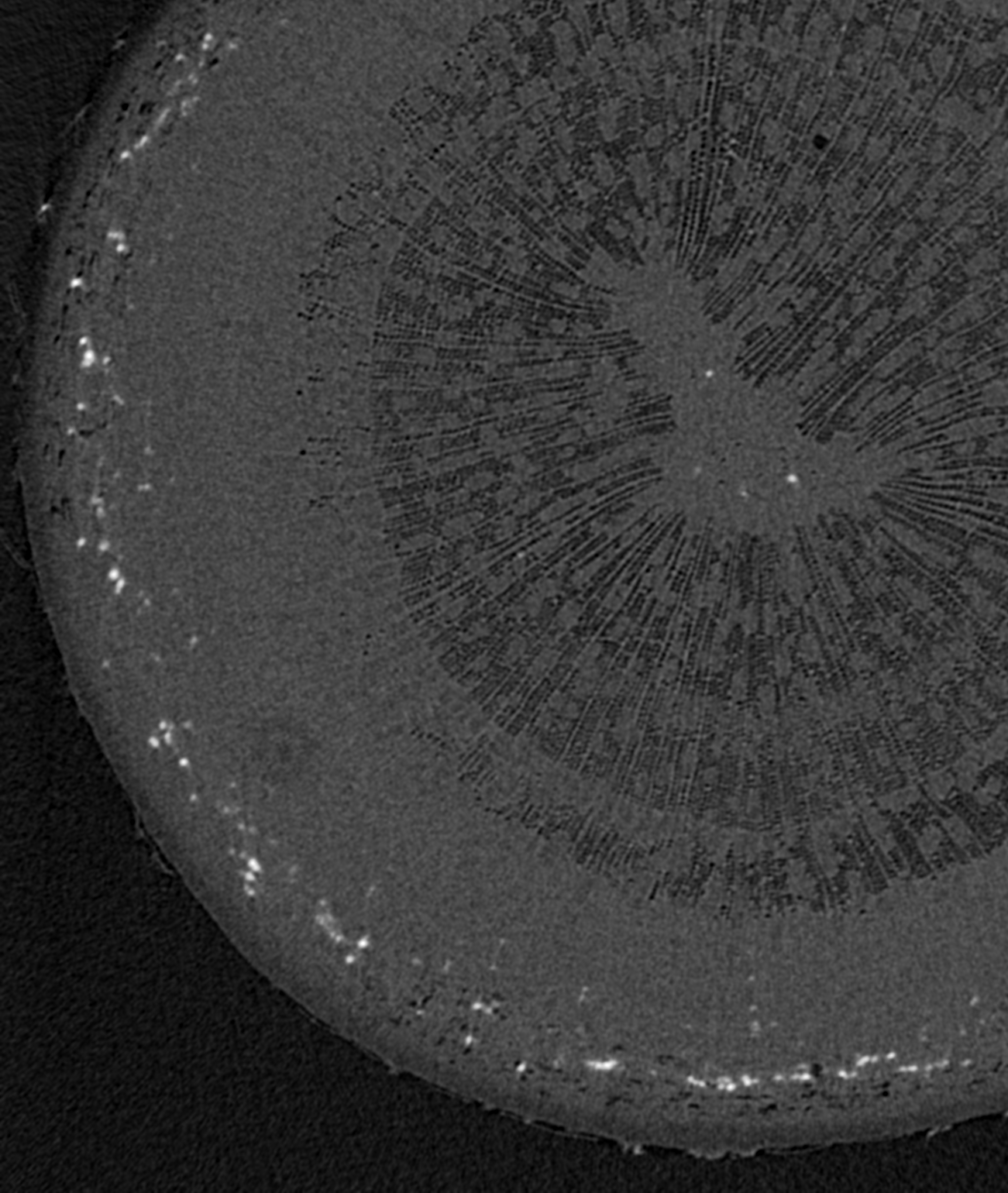

Supplement: Additional file 7 — The original size bilateral filtered data of the cross-section in Figure 4a. The data is grayscale in unsigned 16-bit integer format. Pixel size is 2.00 × 2.00 μm2. [file 1746-4811-9-11-S7.tiff]

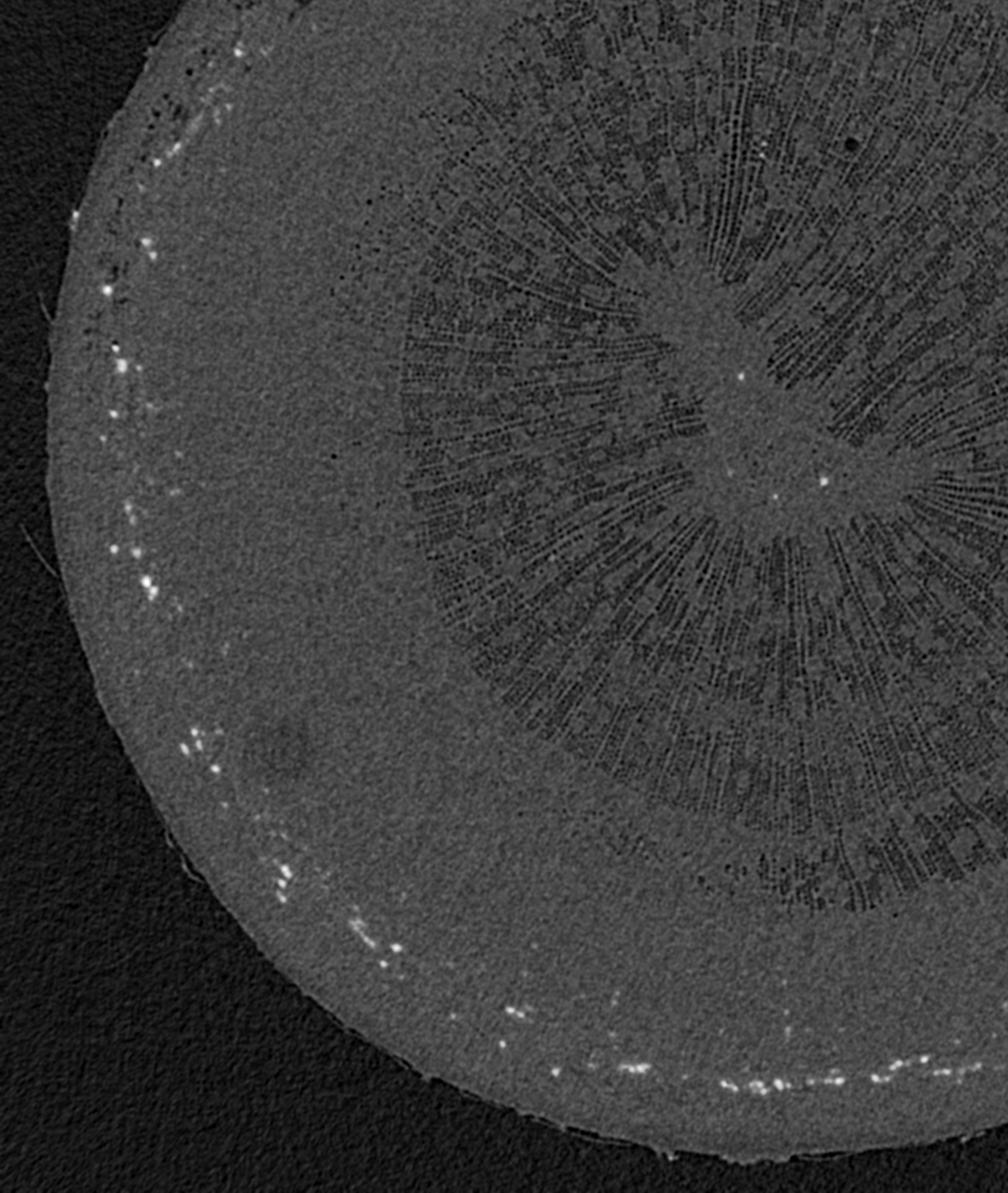

Supplement: Additional file 8 — The original size bilateral filtered data of the cross-section in Figure 4b. The data is grayscale in unsigned 16-bit integer format. Pixel size is 2.00 × 2.00 μm2. [file 1746-4811-9-11-S8.tiff]

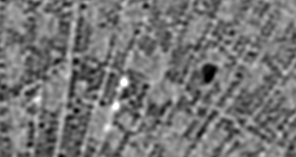

Supplement: Additional file 9 — The original size bilateral filtered data of the cross-section in Figure 5a. The data is grayscale in unsigned 16-bit integer format. Pixel size is 2.00 × 2.00 μm2. [file 1746-4811-9-11-S9.tiff]

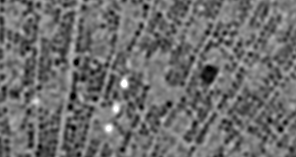

Supplement: Additional file 10 — The original size bilateral filtered data of the cross-section in Figure 5b. The data is grayscale in unsigned 16-bit integer format. Pixel size is 2.00 × 2.00 μm2. [file 1746-4811-9-11-S10.tiff]

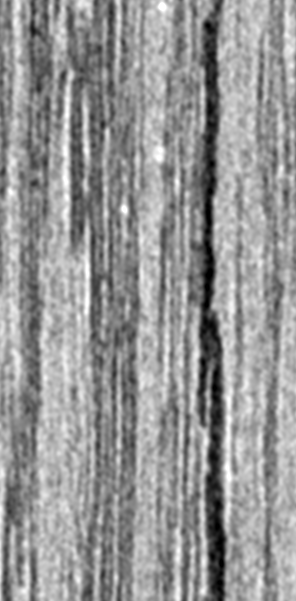

Supplement: Additional file 11 — The original size bilateral filtered data of the vertical cross-section in Figure 5c. The data is grayscale in unsigned 16-bit integer format. Pixel size is 2.00 × 2.00 μm2. [file 1746-4811-9-11-S11.tiff]

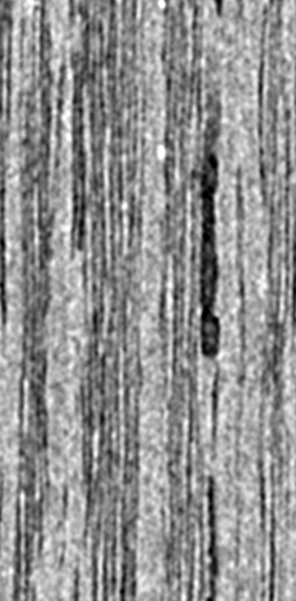

Supplement: Additional file 12 — The original size bilateral filtered data of the vertical cross-section in Figure 5d. The data is grayscale in unsigned 16-bit integer format. Pixel size is 2.00 × 2.00 μm2. [file 1746-4811-9-11-S12.tiff]

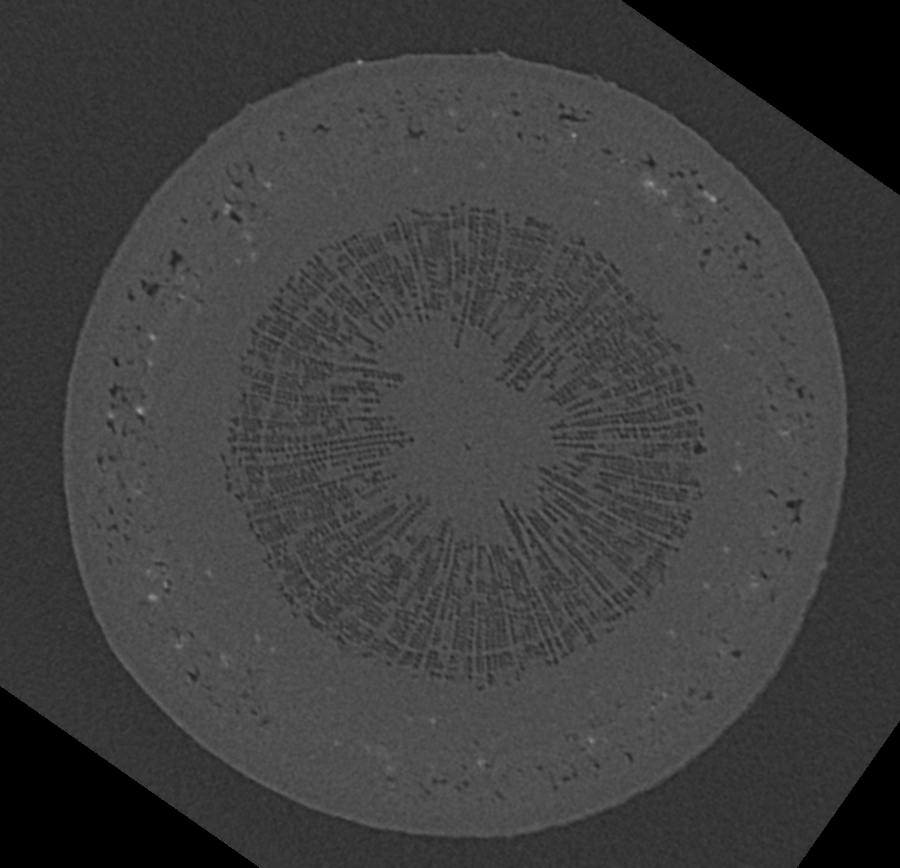

Supplement: Additional file 13 — The original size bilateral filtered data of the cross-section in Figure 6a. The data is grayscale in unsigned 16-bit integer format. Pixel size is 2.50 × 2.50 μm2. [file 1746-4811-9-11-S13.tiff]

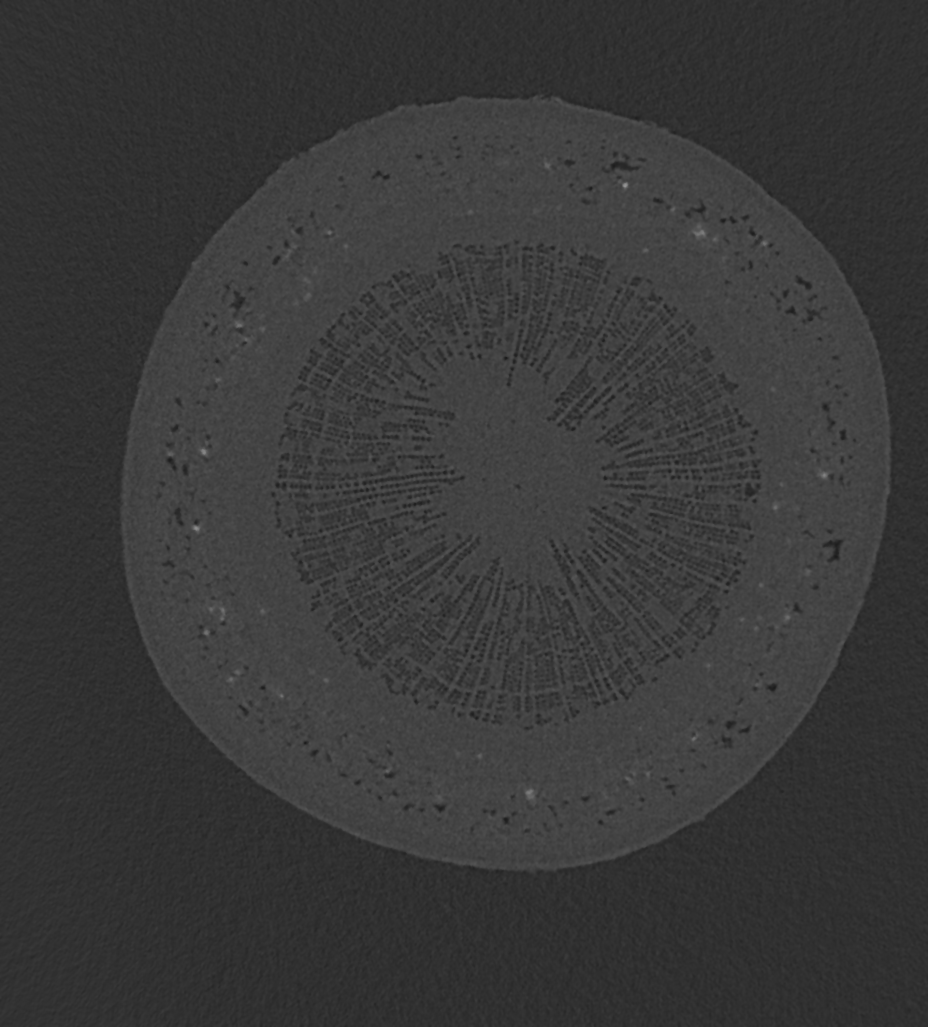

Supplement: Additional file 14 — The original size bilateral filtered data of the cross-section in Figure 6b. The data is grayscale in unsigned 16-bit integer format. Data is resampled to a pixel size of 2.50 × 2.50 μm2, when the original voxel size in the reconstruction was 2.34 × 2.34 × 2.34 μm3. [file 1746-4811-9-11-S14.tiff]

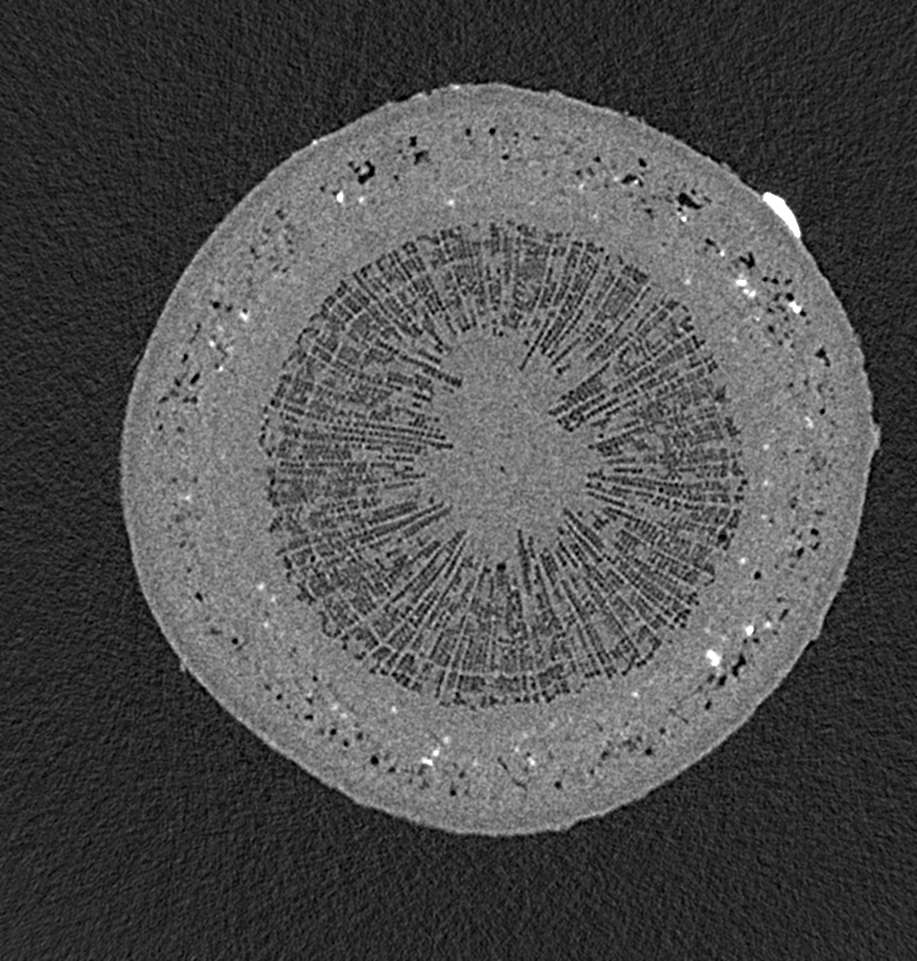

Supplement: Additional file 15 — The original size bilateral filtered data of the cross-section in Figure 6c. The data is grayscale in unsigned 16-bit integer format. Data is resampled to a pixel size of 2.50 × 2.50 μm2, when the original voxel size in the reconstruction was 2.05 × 2.05 × 2.05 μm3. [file 1746-4811-9-11-S15.tiff]

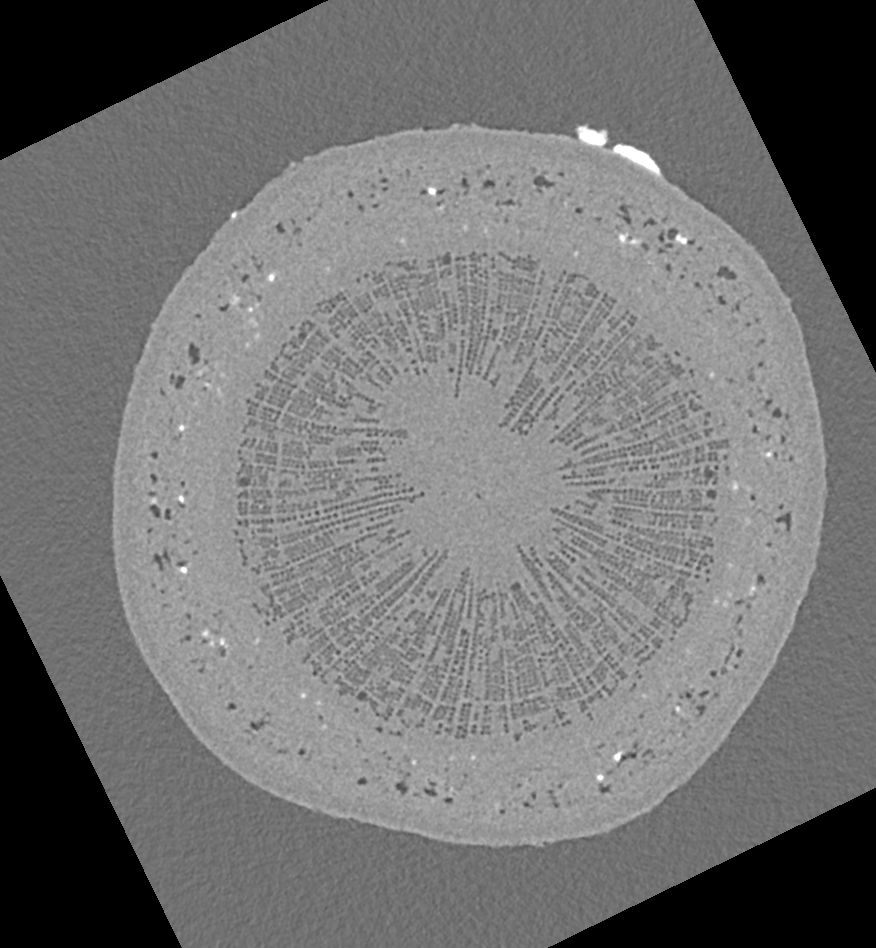

Supplement: Additional file 16 — The original size bilateral filtered data of the cross-section in Figure 6d. The data is grayscale in unsigned 16-bit integer format. Data is resampled to a pixel size of 2.50 × 2.50 μm2, when the original voxel size in the reconstruction was 2.27 × 2.27 × 2.27 μm3. [file 1746-4811-9-11-S16.tiff]

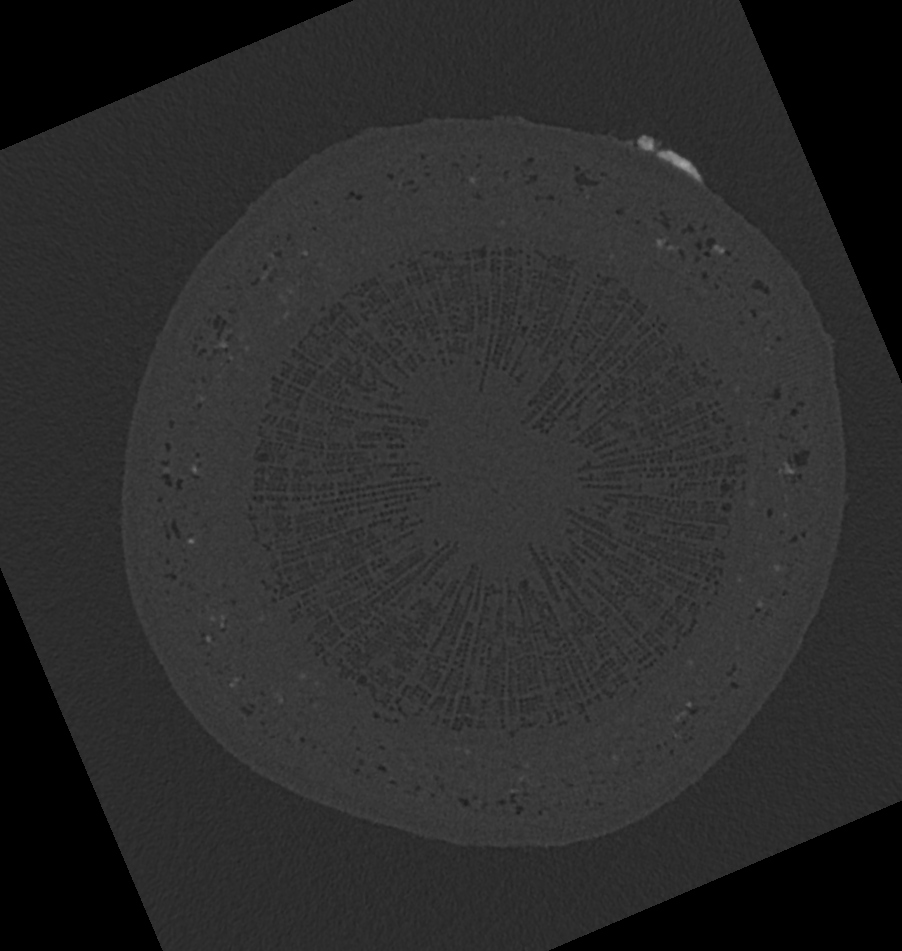

Supplement: Additional file 17 — The original size bilateral filtered data of the cross-section in Figure 6e. The data is grayscale in unsigned 16-bit integer format. Data is resampled to a pixel size of 2.50 × 2.50 μm2, when the original voxel size in the reconstruction was 2.10 × 2.10 × 2.10 μm3. [file 1746-4811-9-11-S17.tiff]

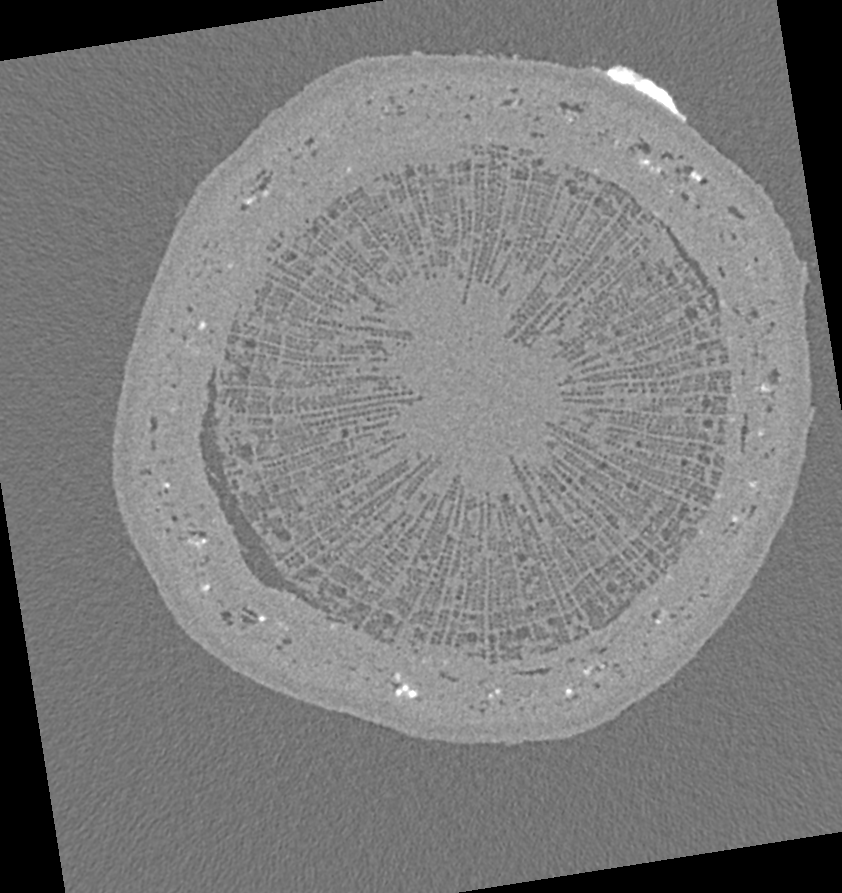

Supplement: Additional file 18 — The original size bilateral filtered data of the cross-section in Figure 6f. The data is grayscale in unsigned 16-bit integer format. Data is resampled to a pixel size of 2.50 × 2.50 μm2, when the original voxel size in the reconstruction was 2.10 × 2.10 × 2.10 μm3. [file 1746-4811-9-11-S18.tiff]

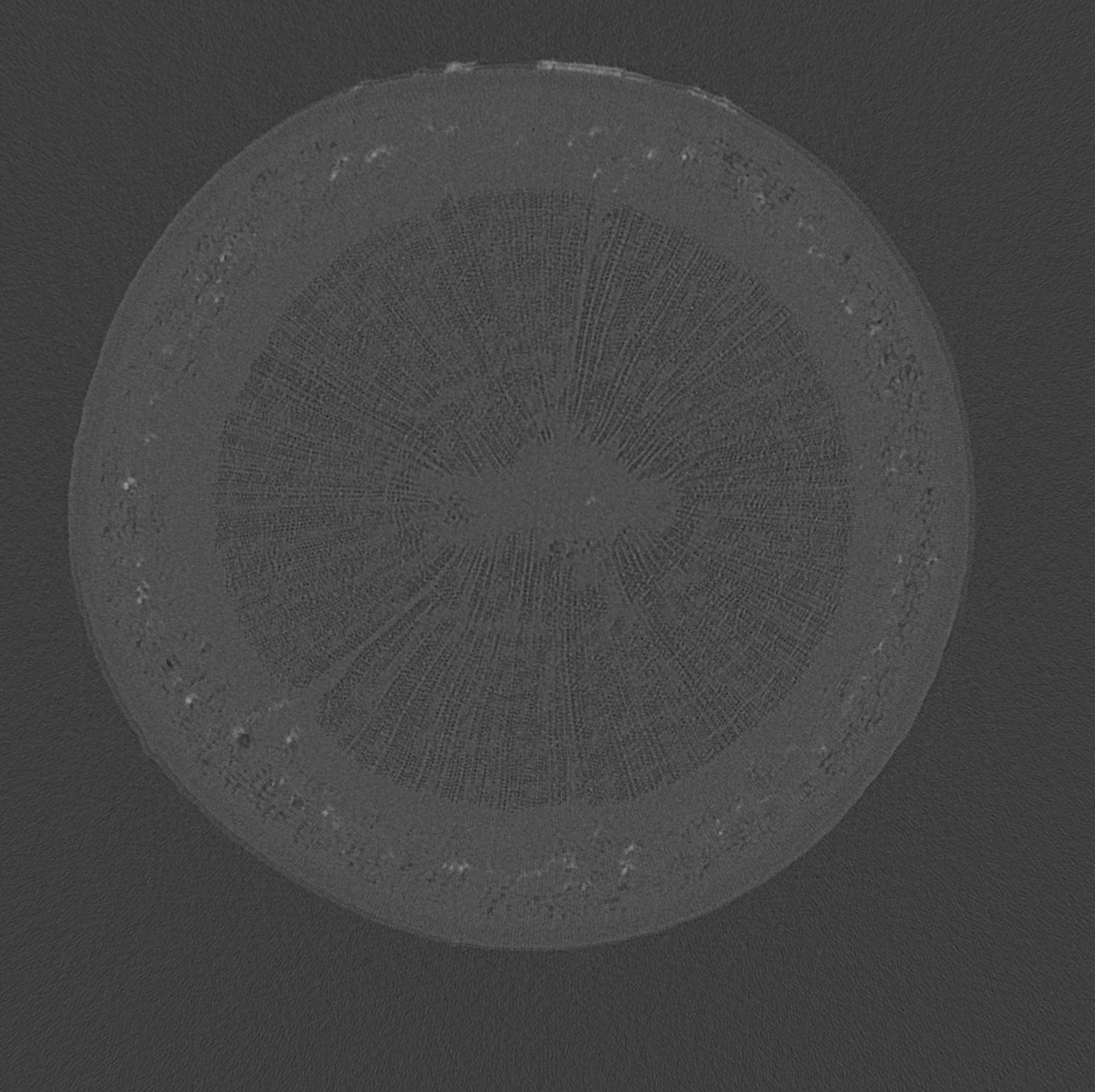

Supplement: Additional file 19 — The original size bilateral filtered data of the cross-section in Figure 7a. The data is grayscale in unsigned 16-bit integer format. Data is resampled to a pixel size of 2.30 × 2.30 μm2, when the original voxel size in the reconstruction was 2.23 × 2.23 × 2.23 μm3. [file 1746-4811-9-11-S19.tiff]

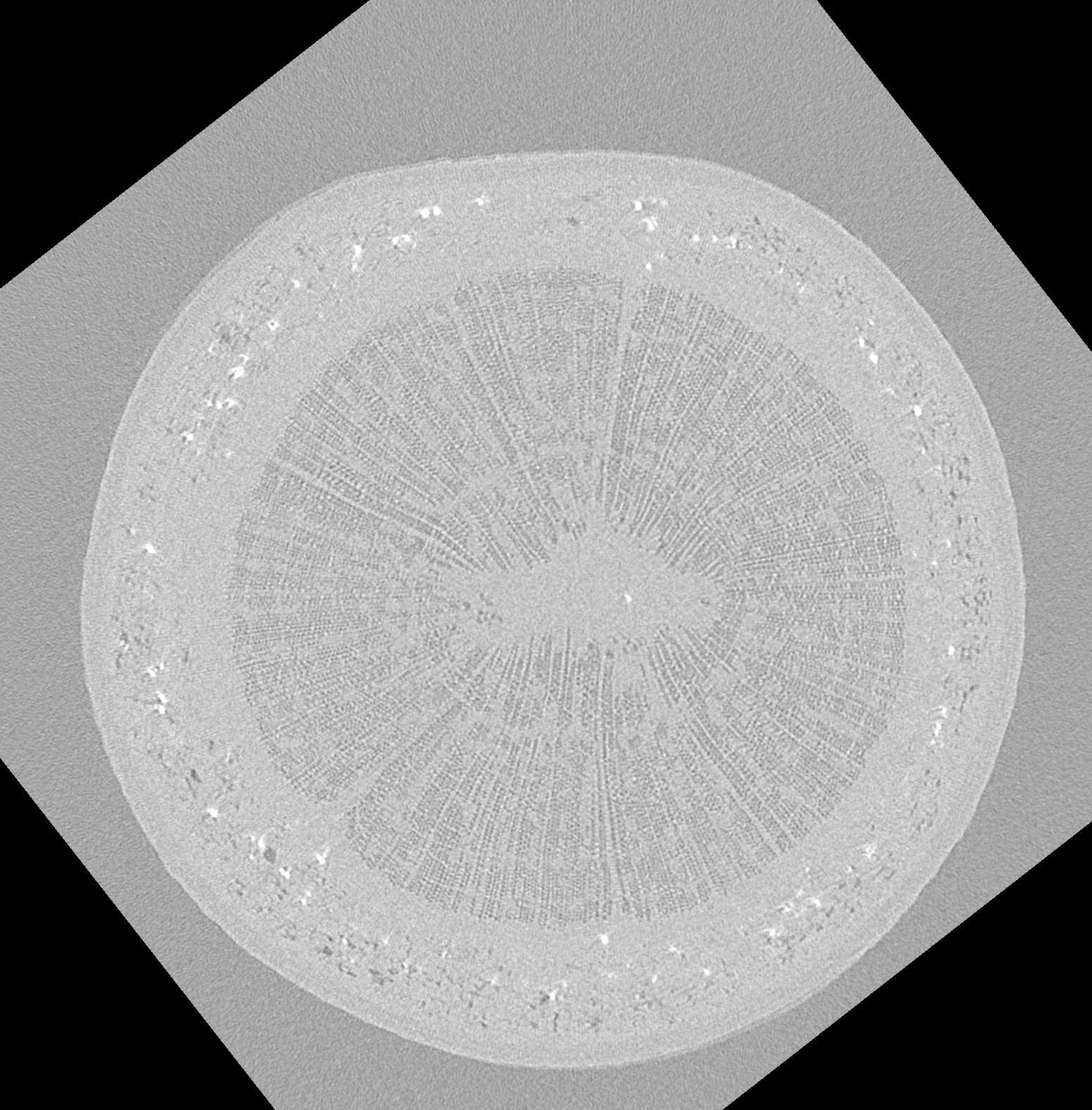

Supplement: Additional file 20 — The original size bilateral filtered data of the cross-section in Figure 7b. The data is grayscale in unsigned 16-bit integer format. Data is resampled to a pixel size of 2.30 × 2.30 μm2, when the original voxel size in the reconstruction was 1.98 × 1.98 × 1.98 μm3. [file 1746-4811-9-11-S20.tiff]

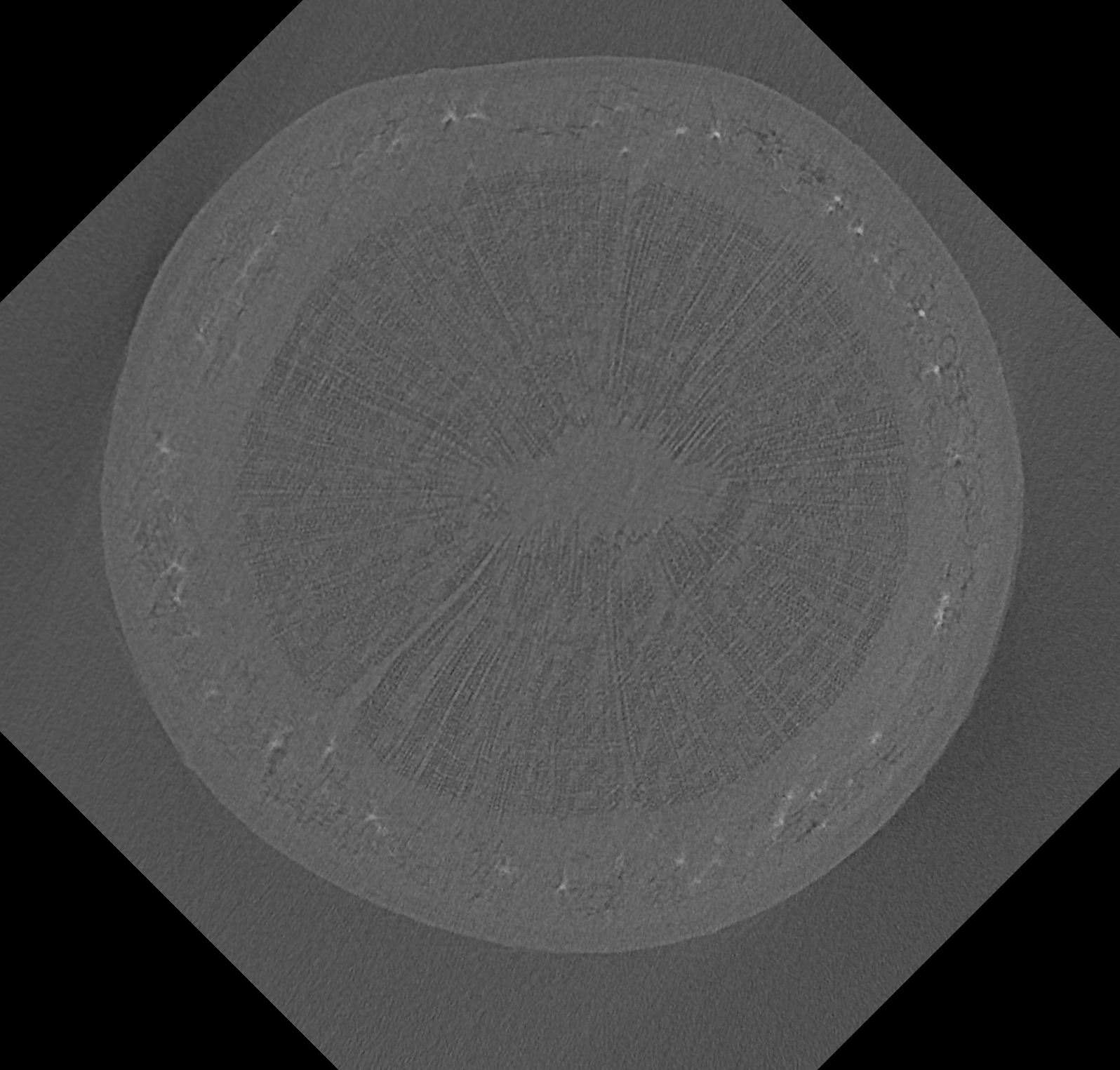

Supplement: Additional file 21 — The original size bilateral filtered data of the cross-section in Figure 7c. The data is grayscale in unsigned 16-bit integer format. Data is resampled to a pixel size of 2.30 × 2.30 μm2, when the original voxel size in the reconstruction was 2.26 × 2.26 × 2.26 μm3. [file 1746-4811-9-11-S21.tiff]

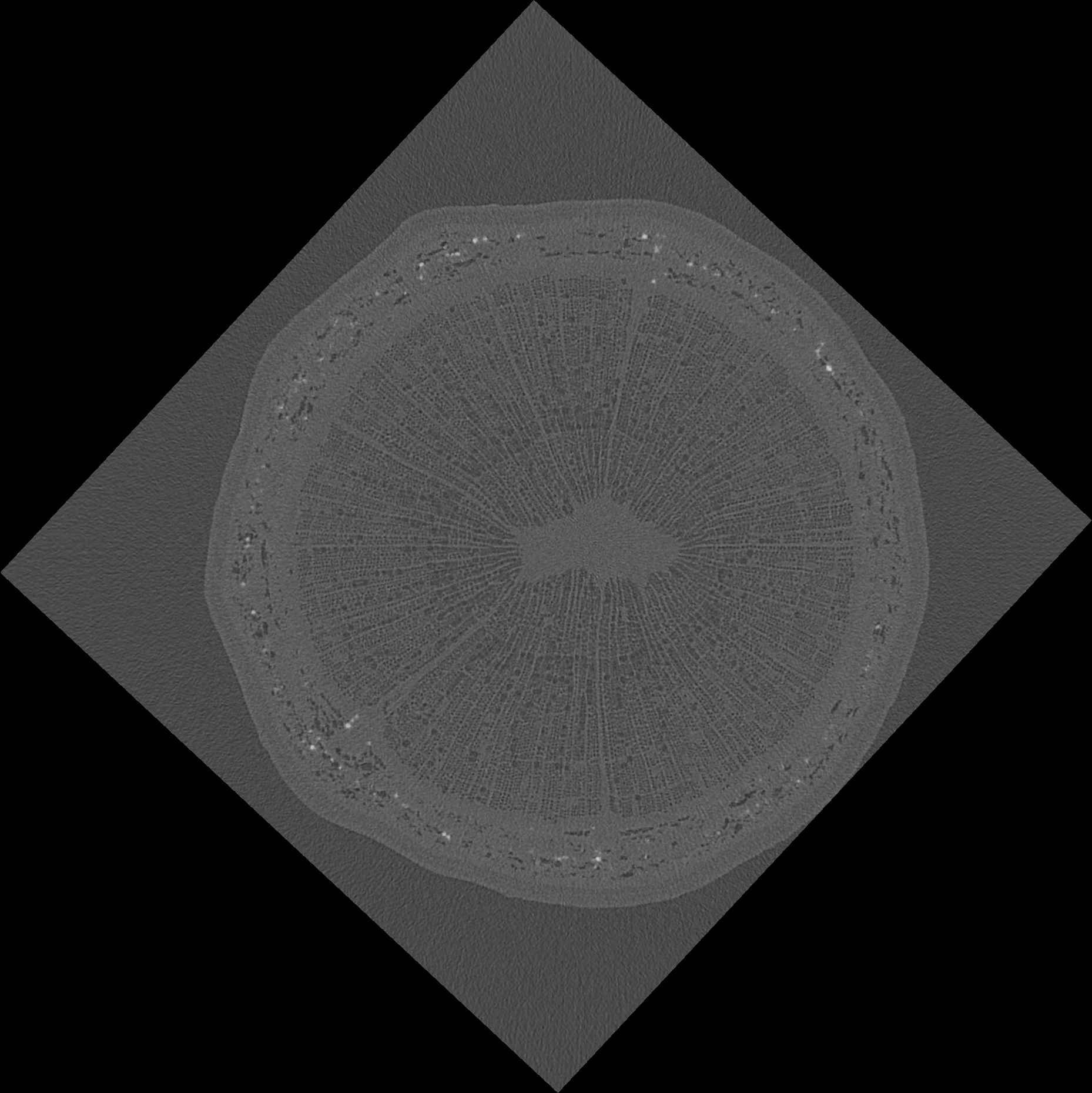

Supplement: Additional file 22 — The original size bilateral filtered data of the cross-section in Figure 7d. The data is grayscale in unsigned 16-bit integer format. Data is resampled to a pixel size of 2.30 × 2.30 μm2, when the original voxel size in the reconstruction was 2.06 × 2.06 × 2.06 μm3. [file 1746-4811-9-11-S22.tiff]

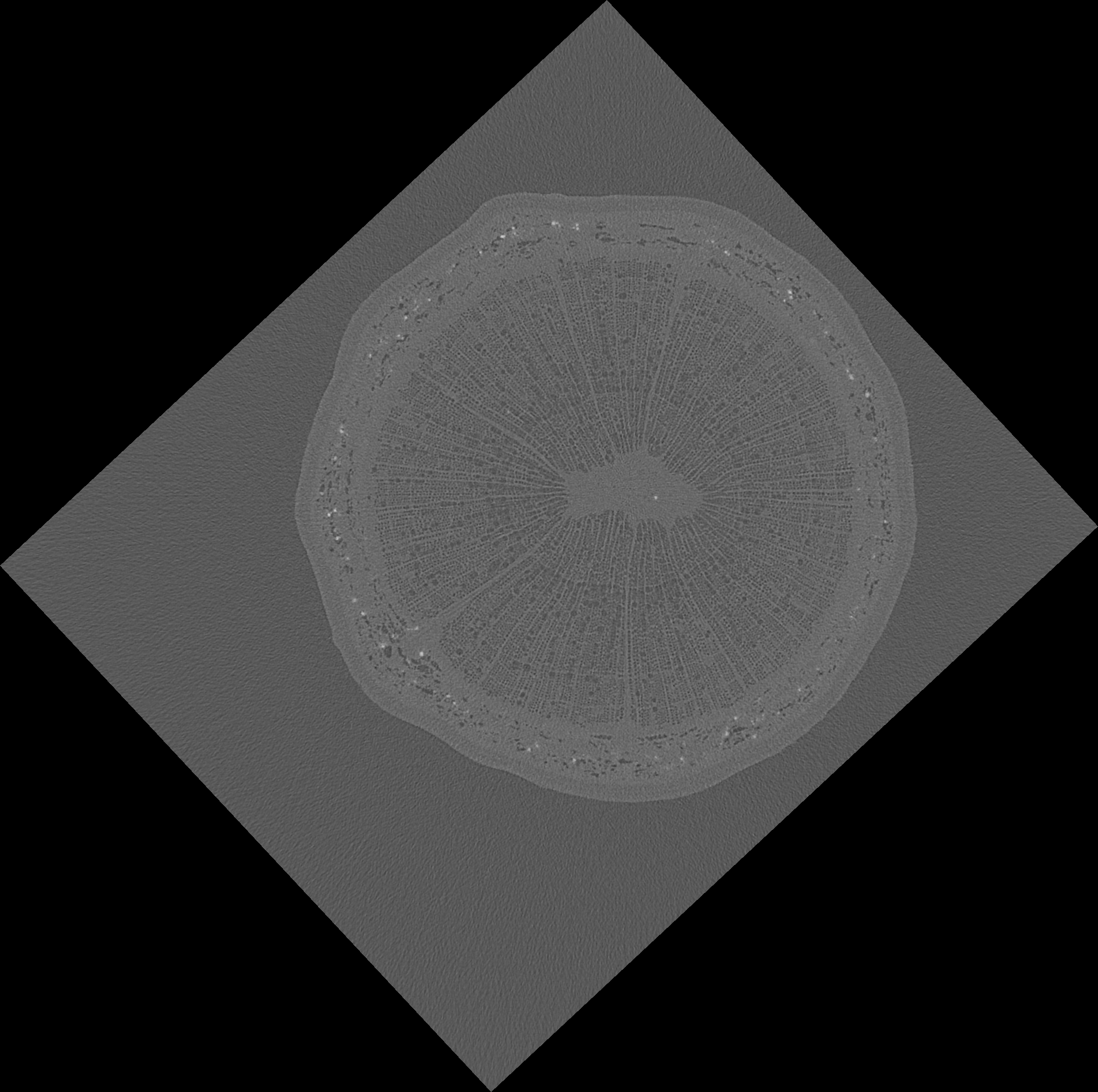

Supplement: Additional file 23 — The original size bilateral filtered data of the cross-section in Figure 7e. The data is grayscale in unsigned 16-bit integer format. Data is resampled to a pixel size of 2.30 × 2.30 μm2, when the original voxel size in the reconstruction was 2.14 × 2.14 × 2.14 μm3. [file 1746-4811-9-11-S23.tiff]

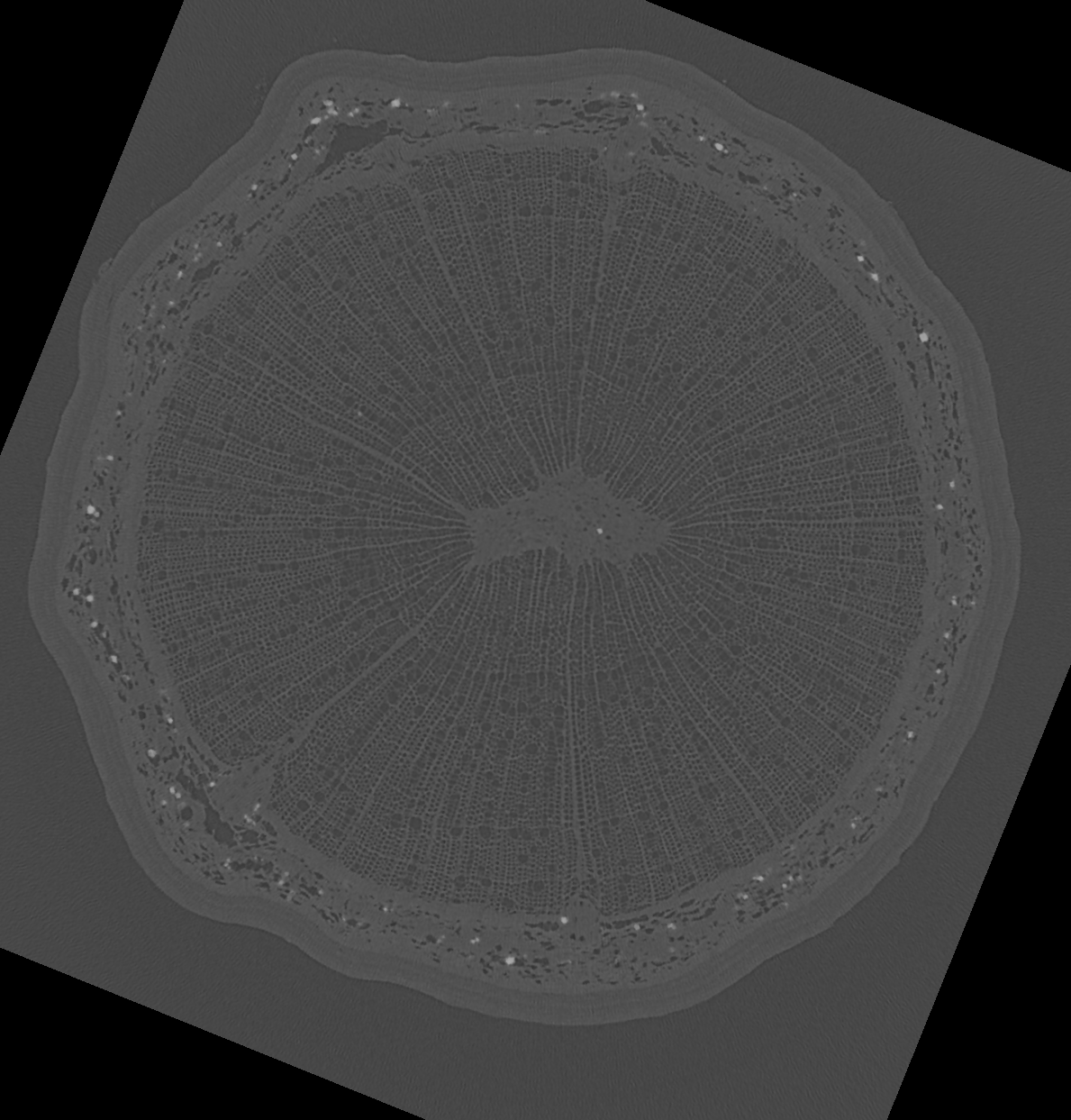

Supplement: Additional file 24 — The original size bilateral filtered data of the cross-section in Figure 7f. The data is grayscale in unsigned 16-bit integer format. Data is resampled to a pixel size of 2.30 × 2.30 μm2, when the original voxel size in the reconstruction was 1.28 × 1.28 × 1.28 μm3. [file 1746-4811-9-11-S24.tiff]

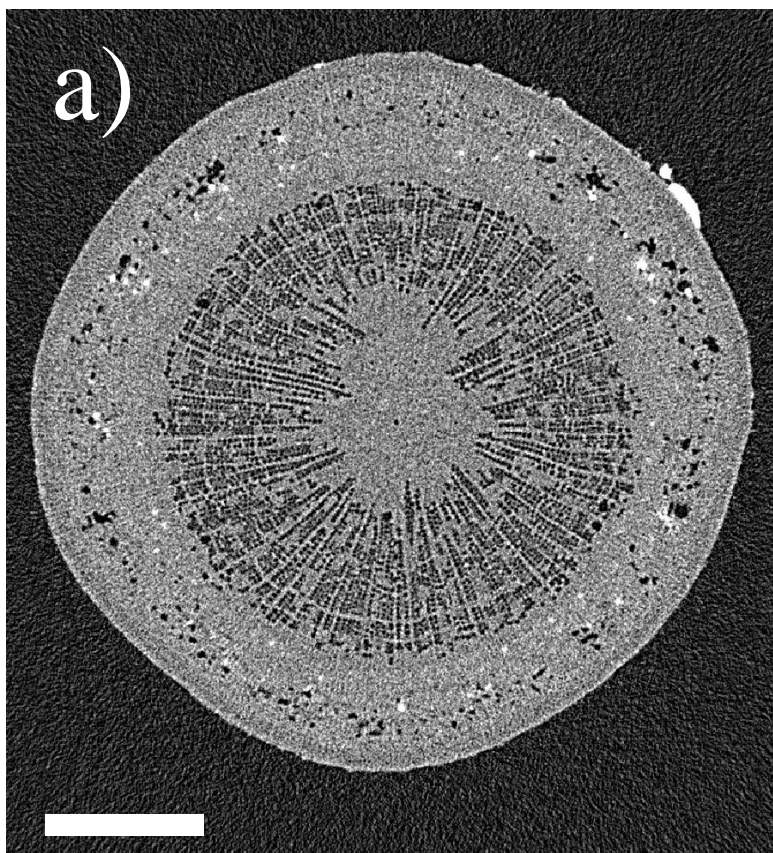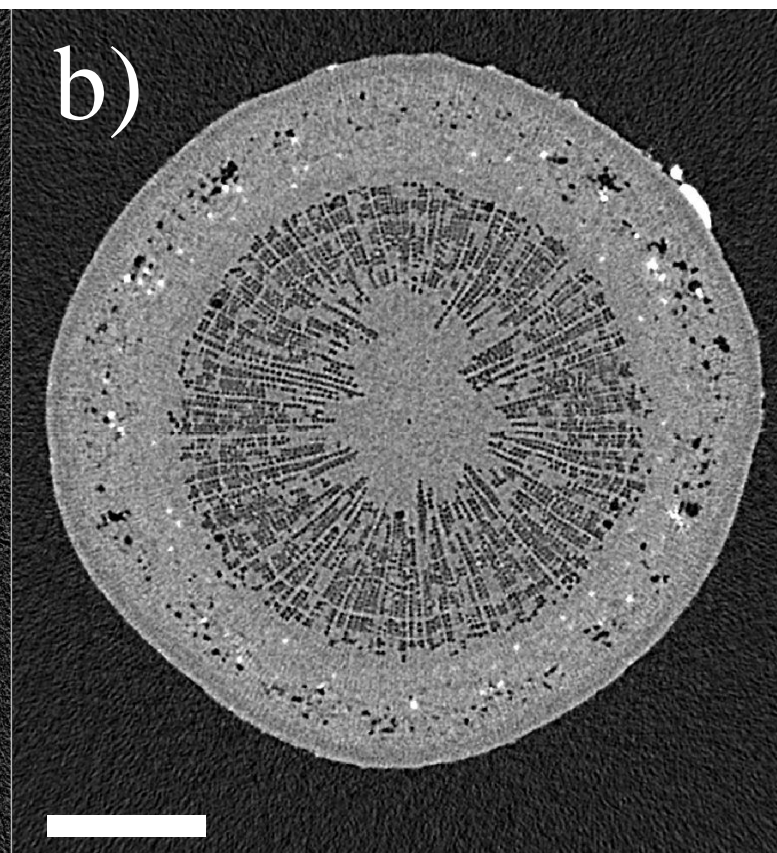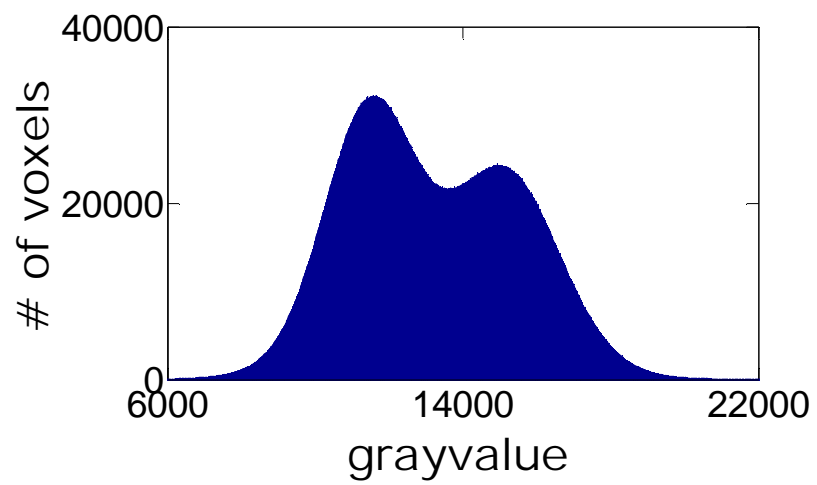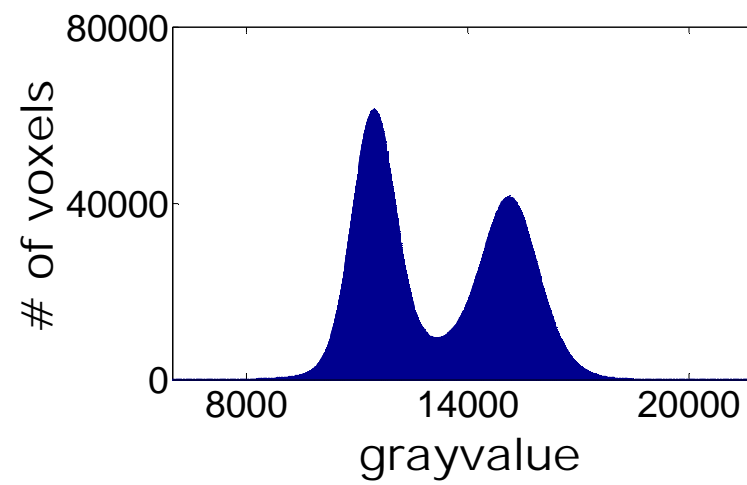

Supplement: Additional file 25 — Left: cross-section of the original XMT data used to produce Figure 1. Right: the same data after bilateral filtering to reduce noise (same as Figure 1a). Scale bars are 400 μm. Histograms of the voxel values are also shown; brightness and contrast of the images are set to show a grayvalue of 10200 as black and a grayvalue of 19000 as white. [file 1746-4811-9-11-S25.pdf]
